# Functional performance tests, clinical measurements, and patient-reported outcome measures do not correlate as outcomes 1 year after anterior cruciate ligament reconstruction

**DOI:** 10.1007/s00167-023-07648-w

**Published:** 2023-11-10

**Authors:** Mustafa Al-Gburi, Jakob Bredahl Kristiansen, Karl Bang Christensen, Michael Rindom Krogsgaard

**Affiliations:** 1 Sections for Sports Traumatology, Department of Orthopedic Surgery, Copenhagen University Hospital Bispebjerg, Copenhagen Ø, Denmark; 2 Department of Physiotherapy, Copenhagen University Hospital Bispebjerg, Copenhagen Ø, Denmark; 3 Section for Biostatistics, Copenhagen University, Copenhagen K, Denmark

**Keywords:** Anterior cruciate ligament reconstruction, Outcome, Patient reported outcome measures, Functional tests, Laxity, Correlations

## Abstract

**Purpose:**

The results after anterior cruciate ligament reconstruction (ACLR) are evaluated by laxity measures, functional tests, and patients’ perception by patient-reported outcome measures (PROMs). It is not known, if one of these evaluation instruments is representative or if outcome scores from all must be reported to obtain a full evaluation of the condition. The aim was to study the correlations between these three types of outcomes 1 year after primary ACLR.

**Method:**

All adult patients (range 18–45 years) who had an ACLR between 1.1.2019 and 31.12.2021 were offered 1-year follow-up by an independent observer. Preoperative information about knee laxity and peroperative information about the condition of menisci and cartilage were registered. At 1-year follow-up clinical and instrumented knee stability and function assessed by four different hop tests were registered. Patients completed four PROMs (the Subjective International Knee Documentation Committee (IKDC) score, the Knee Numeric-Entity Evaluation Score (KNEES-ACL), the Knee injury and Osteoarthritis Outcome Score (KOOS) and the Lysholm score) and Tegner activity scale and answered anchor questions regarding satisfaction and willingness to repeat the operation.

**Results:**

A total of 190 adults attended the 1-year follow-up and 151 had all assessments. There were only a few positive and weak correlations between performance tests and PROMS and between clinical measurements and PROMS (*r* = 0.00–0.38), and the majority were of negligible strength. Tegner score had in general the highest correlation (low to moderate). The highest correlation was 0.53 (moderate) between the anchor question about patient satisfaction and Lysholm/IKDC scores. There was no difference in the correlations depending on meniscal condition.

**Conclusions:**

In ACLR patients there was no clinically relevant correlation between scores obtained by PROMs, a battery of functional performance tests and instrumented laxity of the knee at 1-year follow-up. Therefore, one type of outcome cannot represent the others. This is an argument for always to include and report all three types of outcomes, and conclusions based on one type of outcome may not be sufficient.

**Level of evidence:**

II.

**Supplementary Information:**

The online version contains supplementary material available at 10.1007/s00167-023-07648-w.

## Introduction

The results after anterior cruciate ligament reconstruction (ACLR) can be evaluated by scores from patient-reported outcome measures (PROMs), measures of activity level, clinical and instrumented joint laxity, muscle strength, balance, and functional tests [6, 28].

PROM scores are often regarded as the most important outcomes in clinical settings [10, 27], as they are assumed to express the patients’ subjective description of the condition [20]. Dynamic performance tests can be used to assess when it is safe to return to sport [14, 15], and clinical and instrumented laxity measures can indicate whether the intention to obtain a normal mechanical knee laxity has been reached [1].

Functional tests are time-consuming and relatively complex to perform, [14, 15, 17, 22, 23], which may be the reason why they are rarely reported in the literature [1]. Examples of suggested criteria for return to sport are a limb symmetry index (LSI) > 90% on isokinetic and hop tests, and subjective IKDC scores within the 15th percentile of healthy subjects [11].

Optimally, function should be positively related to knee laxity, and patients with a good function should show high PROM scores. In that case, one type of outcome could be representative of all, but there are only two studies on whether such correlations exist. One tested the correlation between the limb symmetry index for the one-leg vertical jump test against the subjective IKDC scores (*r* = 0.26), Tegner score (*r* = 0.64) and the ACL-RSI (Return to Sport after Injury) scale scores (*r* = 0.61) nine months after ACLR in younger, adult male athletes [21]. The other found that in children who had an ACLR 1 year earlier there were no clinically important correlations between scores obtained by Pedi-IKDC and KOOS-Child, laxity measures, knee muscle strength and four functional performance tests [33].

Many musculoskeletal randomized clinical trials report PROM scores as the primary or only outcome, and several important healthcare strategies are based on PROM outcomes. The prerequisite for this is that PROM scores are representative of the outcome of the treatment, even though this is a philosophically/politically point of view and not scientifically based [20]. Therefore, it is of interest to analyse whether this is a reasonable prerequisite for patients treated with ACL reconstruction. It was hypothesized that with an adequate PROM and functional and clinical/instrumented measures obtained by highly experienced and independent observers there would be strong correlations between the different types of measure and that it is sufficient to report only one as representative for them all. The purpose of this study was to evaluate the correlations between three outcomes that are generally regarded as important in relation to these patients: four functional performance tests, clinical and instrumented knee laxity, and PROM scores. The analyses were performed 1 year after ACLR in a consecutive, prospective series of patients.

## Materials and method

The 1-year follow-up has been part of the standard program offered to patients after ACLR at the section for sports traumatology, department of orthopedic surgery at Copenhagen University Hospital Bispebjerg since 2007, and the regional ethical committee stated that ethical permission was not required for this study (ref. nr. F-23055524). The patients gave written consent to have their data registered, and permission to store data for this study was obtained. The study was performed according to the STROBE checklist for cohort studies.

**Fig. 1:**
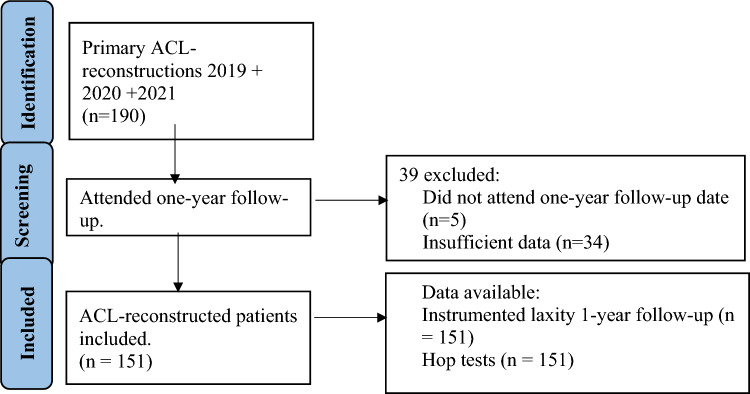
The study flowchart summarizing screening, inclusion and exclusion of the patients

Between January 1, 2019, and December 31, 2021, 190 patients had a primary ACLR (patients with revision ACLR, multiple ligament injuries or pathology of the contralateral knee (including ACLR) were not included). Of these, 151 attended the 1-year follow-up, and they were included in this study (Fig. 1). Meniscal pathology or cartilage defects was not an exclusion criteria. All patients were seen by a physical therapist before discharge after the operation and were given a standard rehabilitation program. Almost all patients attended supervised rehabilitation, either free of charge in a municipal physiotherapy clinic (typically for three months) or with a physical therapist affiliated with a sports club or to a private clinic. Braces were not used, except in cases with meniscal refixation, in which case full weight bearing was allowed but with flexion restricted to 40° for 2 weeks and to 90° for the subsequent 2 weeks.

### Surgical techniques

For all ACLRs, the drill tunnels were positioned at the anatomical insertion sites of ACL. Drilling of the femoral tunnel was through the anteromedial portal, inside-out, and of the tibial portal outside-in. Standard grafts were doubled hamstring tendons, and they were fixed by EndoButton (Smith & Nephew) on the femur and by Intrafix with a sheath (DePuy) on tibia. Patellar tendon grafts and quadriceps tendon grafts were fixed with Peek screws (Arthrex), and iliotibal tract grafts were fixed with TightRope (Arthrex) at tibia and a peek screw on femur. The grafts that had been used are listed in Table 1. Meniscal lesions were either resected or repaired using FastFix (Smith & Nephew) (Table 2). In this cohort two patients had cartilage defects (one patient with a defect in the medial femoral cartilage and one in the lateral femoral cartilage), and both were left untouched.

**Table 1:** Grafts used for reconstruction of the anterior cruciate ligament in the 151 patients included in the study

Graft type	Number of patients
Semi-tendinosus + Gracilis tendons	122
Patellar tendon	25
Iliotibial tract	4

**Table 2:** Details of meniscal injuries and their treatment in 151 patients who had a reconstruction of the anterior cruciate ligament

Type of meniscus injury	Number of patients	Type of treatment
Medial	47	*n* = 39 repair *n* = 7 resection *n* = 1 root insertion
Lateral	20	*n* = 8 repair *n* = 7 resection *n* = 5 root insertion
Medial and lateral	7	*n* = 3 medial and lateral repair *n* = 3 repair of medial meniscus + resection of lateral meniscus *n* = 1 repair of the medial meniscus and root re-insertion of the lateral meniscus

### Clinical evaluation

Information was prospectively registered: Preoperative data on gender, age, results of manual Lachman, drawer- and pivot shift tests, instrumented stability by Rolimeter in 30° of knee flexion, and results of the dial test and MCL/LCL stability tests—all stability tests were performed in both knees. There was peroperative information on concomitant injuries and their treatment. The 1-year follow-up was performed by a physical therapist (one of three) with special experience in knee ligament injury and functional testing. Lachman-, drawer- and pivot shift tests and instrumented stability measurements were performed.

Laxity was measured in mm by the Rolimeter as described by Ganko [9] with the knee in 30° flexion and the patient in the supine position. With the patient fully relaxed, the tibia was pulled anteriorly as far as possible with maximal power. The measurement was performed three times, and the last result was registered. Lachman stability was tested as described in [31], anterior and posterior drawer as in [25], pivot shift as in [8], side-stability as in [24] and dial test as in [24].

### Patient-reported outcome measures (PROMs)

At 1-year follow-up, patients completed four patient-reported outcome measures (PROMs): the Lysholm score, the Subjective IKDC score, the Knee Numeric-Entity Evaluation Score** (**KNEES-ACL) and the Knee injury and Osteoarthritis Outcome Score (KOOS). Activity was assessed by the Tegner Score. Anchor questions were answered: satisfaction with the ACLR, indicated on a 3-point scale (very satisfied, satisfied, not satisfied), and whether the patient would prefer the same treatment if the postoperative result had been known preoperatively (on a 3-point scale: yes, perhaps, no).

The IKDC questionnaire was published in 2001 [16] and it was intended for patients with a variety of knee conditions. It was developed by experts without the involvement of patients, and therefore it has no proven content validity [12, 13]. KOOS was published in 1998 [29] and it was intended for patients with knee injury and osteoarthritis. It is an aggregation of The Western Ontario and McMaster Universities’ Arthritis Index (WOMAC) [3], consisting of 3 domains with 33 items, plus two domains with 9 items developed through interviews with patients with ACL injury or meniscal conditions. WOMAC was developed for patients with end-stage osteoarthritis of the hip or knee and with the involvement of such patients. KOOS has no proven content validity for patients with ACL injury [12, 13]. Also, it has a low degree of construct validity (measurement properties) for patients with ACL injury when evaluated with item response theory models [4, 13, 19]. The Lysholm score was published in 1982 and updated in 1985, and it intended to evaluate knee ligament surgery [30]. It was developed without patient involvement and has no proven content validity [13]. KNEES-ACL was published in 2013 and intended for patients with ACL injury [5]. It was developed by involvement of patients with ACL injury and has good content validity [12, 13]. Construct validity evaluated with item response theory models is good [13].

### Functional performance tests

All tests were conducted 1 year after the operation by a group of trained physiotherapists who had not been involved in the patients’ surgery. The test battery included single leg hop test (SH), triple hop test (TH), 6-m timed hop test (6 m-timed) and cross-over hop test (COH), as described by Noyes et al. [26]. The intra- and inter-rater reliability of these tests is moderate to excellent [18]. The test procedures are described in detail by Warming et al. [32].

After 5 min of warming up, typically on an exercise bike, followed by 2 min of stretching, the patients made three practice trials prior to each test with adequate rest periods between trials to minimize the effects of fatigue. The practice trials allowed the patients to familiarize themselves with the tests and minimized the learning effects. The best trial score of three jumps was utilized for data analysis, indicating the patient’s maximal performance. All tests had to be performed without losing balance and with a secure landing.

During the single-leg hop the patients stood on one limb, hopped as far forward as possible, and landed on the same limb. The distance was recorded with a tape measure, fixed to the floor. As the subject landed, an investigator recorded the distance from the starting position to the heel strike. Each limb was tested three times. To calculate the limb symmetry index, the best result for the involved limb was divided by the best result for the non-involved limb, and the result was multiplied by 100. A similar procedure was conducted with the triple hop test (to jump as far as possible on a single leg three consecutive times), and the longest distance for each leg was used to calculate limb symmetry.

The single-leg timed hop test was performed over a distance of 6 m with the patients jumping as fast as possible on a single leg. The patients were encouraged to use large, forceful one-legged hopping motions to propel their body forward. The best time of three attempts was chosen for each limb and used for limb symmetry calculation.

The single-leg cross-over triple hop for distance test was performed on a test ground with a 15-cm broad, 6 m long marking strip on the floor. Each patient hopped three consecutive times on one foot, crossing the marking strip during each hop. Each subject was encouraged to jump as far forward as possible during each hop. The total hop distance was measured for each of the three trials, and the best result was used for the calculation of limb symmetry.

After the calculation of the limb symmetry indexes for each of the four hop tests, a composite symmetry index was calculated for the individual patients as the mean of the four indexes.

### Statistical analysis

A comparison was evaluated through Pearson correlations and a 95% confidence interval (CI) computed based on Fisher’s z-transformation. The KOOS and KNEES-ACL domain scores were calculated as raw scores. The strength of the correlations was interpreted as: 0.00–0.30 negligible, 0.30–0.50 low, 0.50–0.70 moderate, 0.70–0.90 high and 0.90–1.00 very high. With *α* = 0.05, *β* = 0.20 and the lowest correlation we regarded as relevant being 0.25 (high in the negligible category and meaning that 6.25% of the changes in one outcome can explain changes in the other outcome) we needed 123 patients in the study [15].

Calculations were performed in SAS.

To evaluate whether the presence of meniscal injury affected the correlations, the two groups [no meniscal pathology (*n* = 77) and meniscal pathology (*n* = 74)] were also analysed separately.

## Results

**Fig. 2:**
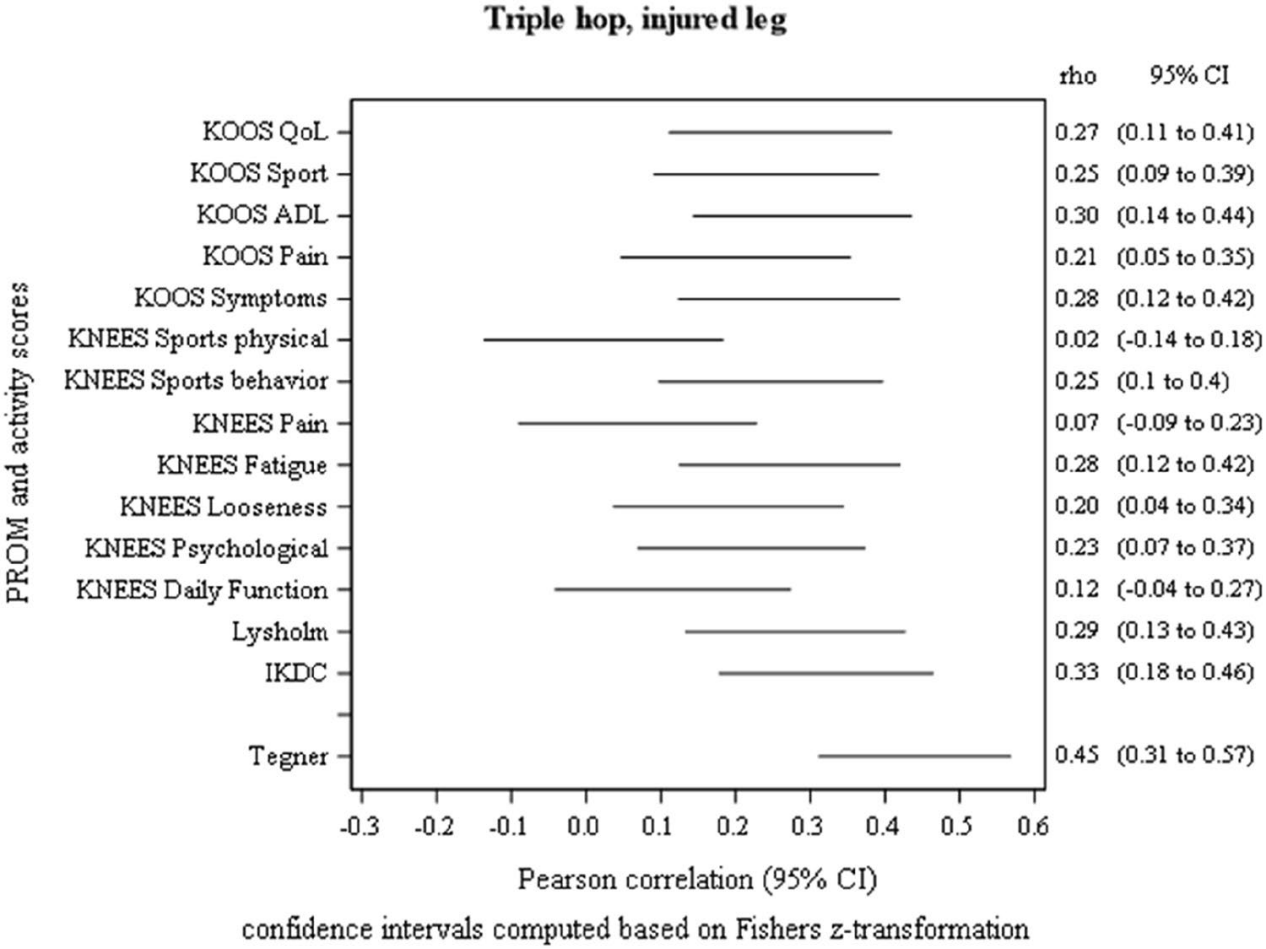
Plot showing the 95% confidence intervals for the Pearson correlations between triple hop test (cm) and scores from KOOS, KNEES-ACL, IKDC, Lysholm Score and Tegner Activity Score in 151 patients at 1-year follow-up after reconstruction of the anterior cruciate ligament

**Fig. 3:**
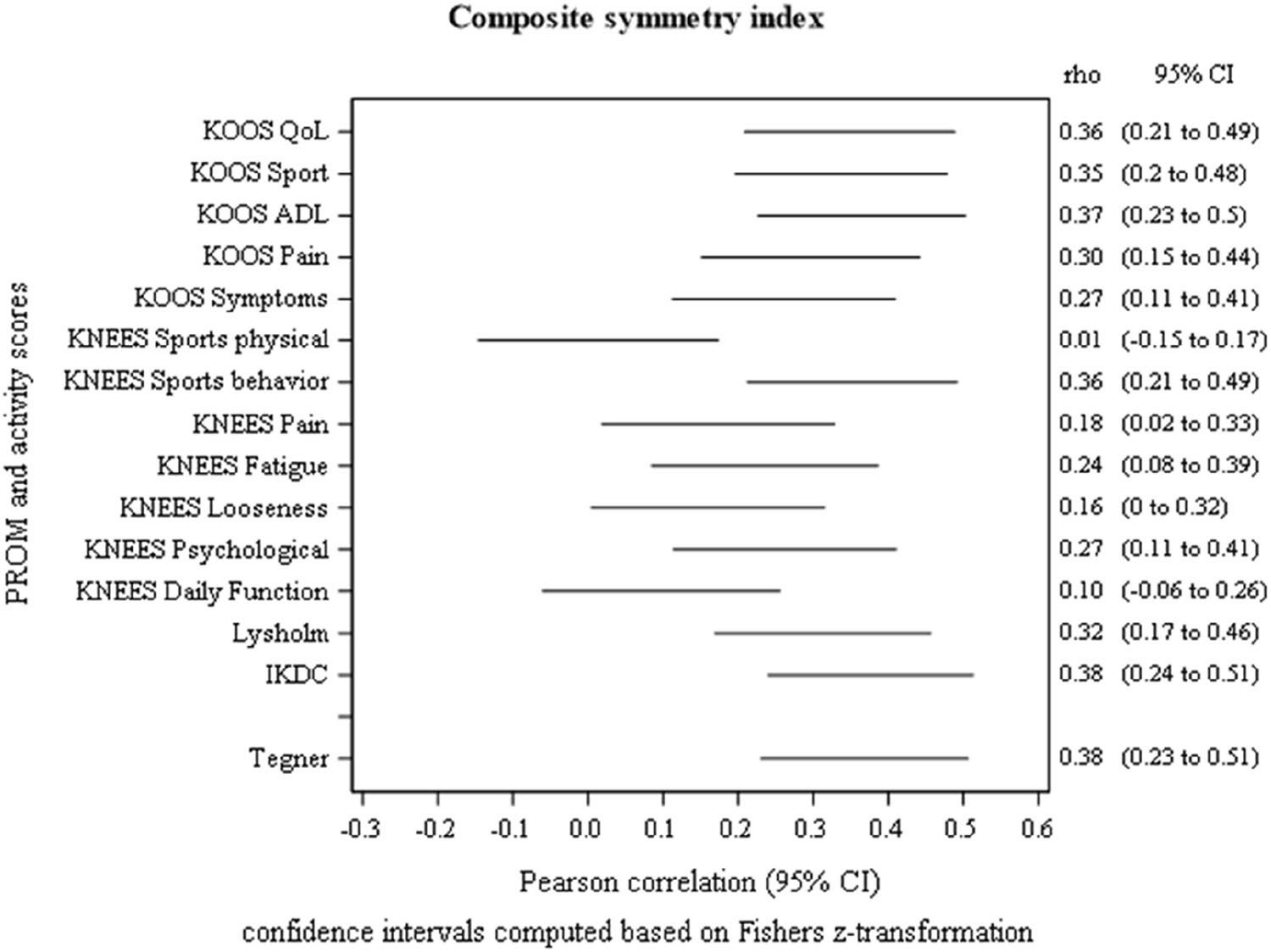
Plot showing the 95% confidence intervals for the Pearson correlations between composite symmetry index (%) and scores from KOOS, KNEES-ACL, IKDC, Lysholm Score and Tegner Activity Score in 151 patients at 1-year follow-up after reconstruction of the anterior cruciate ligament

**Fig. 4:**
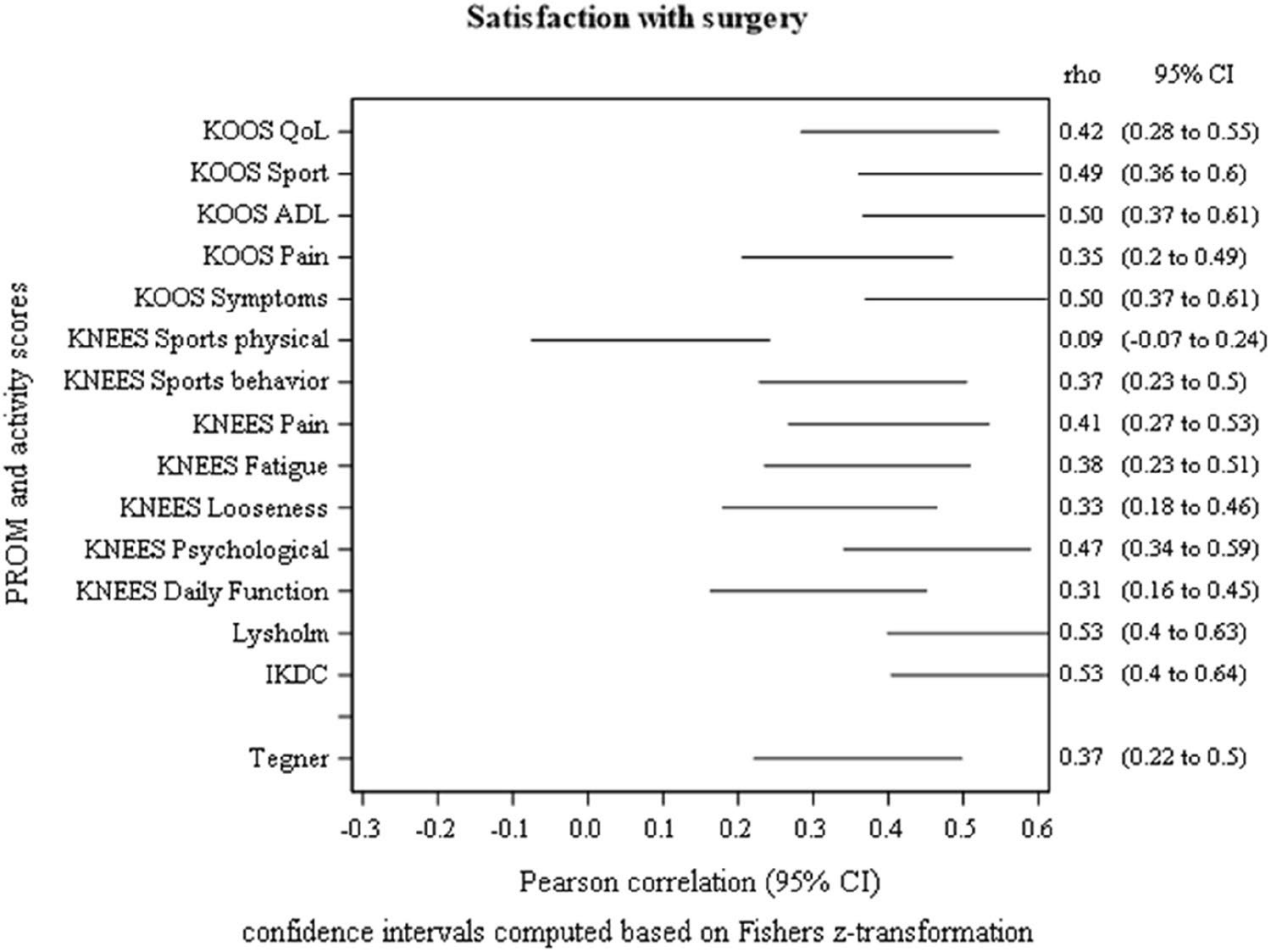
Plot showing the 95% confidence intervals for the Pearson correlations between satisfaction with surgery and scores from KOOS, KNEES-ACL, IKDC, Lysholm Score and Tegner Activity Score in 151 patients at 1-year follow-up after reconstruction of the anterior cruciate ligament

All 151 patients were included in the analysis. There were 65 (43%) women and 86 (57%) men. The mean age was 31 years (18–45 years). Spearman correlations calculated between the PROM score (the Lysholm score, IKDC-score, and each domain score in KNEES-ACL and KOOS), Tegner score, Rolimeter measures (injured side minus un-injured side) and the two anchor questions against each of the four hop tests are reported in the supplementary material S1 as tables and plots. Tegner score had in general the highest correlation (low to moderate), while most other correlations were negligible or low. The three analyses in which there was at least one correlation > 0.30 (except for Tegner score) are shown graphically in Figs. 2, 3 and 4. The highest correlation was 0.53 (moderate) between patient satisfaction and Lysholm and IKDC scores. Overall, PROMs correlated best with satisfaction.

Table 3 shows the median (with a range in parentheses) correlations for each variable compared to PROM/Tegner scores in, respectively, all patients, in patients with meniscal injury and in patients without meniscal injury. There was no difference in the correlations depending on meniscal pathology.

**Table 3:** Median correlation coefficient for each hop test, Rolimeter difference, satisfaction with surgery and willingness to repeat surgery compared to PROM-scores (KNEES-ACL, IKDC, KOOS and Lysholm) and Tegner (activity) score in 151 patients at 1-year follow-up after reconstruction of the anterior cruciate ligament

Variable	Total (*n* = 151)	Meniscal injury (*n* = 74)	No meniscal injury (*n* = 77)
Single hop, injured leg	0.21 (0.00–0.45)	0.21 (– 0.01 to 0.53)	0.26 (0.12–0.38)
6 m timed hop, injured leg	0.03 (– 0.04 to 0.08)	0.10 (– 0.08 to 0.18)	– 0.04 (– 0.14 to 0.12)
Triple hop, injured leg	0.25 (0.02–0.45)	0.26 (0.02–0.49)	0.20 (– 0.03 to 0.42)
Cross over hop, injured leg	0.11 (– 0.06 to 0.39)	0.11 (– 0.18 to 0.37)	0.21 (– 0.05 to 0.42)
Composite symmetry index	0.30 (0.01–0.38)	0.36 (– 0.08 to 0.47)	0.23 (0.09–0.37)
Rolimeter, injured minus non-injured knee	0.08 (– 0.15 to 0.19)	0.08 (– 0.06 to 0.26)	0.08 (– 0.15 to 0.19)
Satisfaction with surgery	0.41 (0.09–0.53)	0.46 (0.13–0.57)	0.37 (0.04–0.56)
Willingness to repeat surgery	0.08 (– 0.06–0.11)	0.10 (– 0.09 to 0.21)	– 0.03 (– 0.13 to 0.10)

Figures as median (range)

Figures 2, 3 and 4: Plots for the three tests with one or several correlation coefficients > 0.30 (except for Tegner score)

## Discussion

This study showed that PROM scores do not correlate with functional or stability tests at 1-year follow-up after ACLR. Therefore, one outcome is not representative for the condition, and the study hypothesis was rejected. The low correlations demonstrated in the current study are comparable to what has been found in children with ACL injury [33]. In a group of 75 athletes who were tested in relation to return to sport 9 months after ACLR there was a negligible correlation between limb symmetry index of single leg vertical jump height and IKDC score (*r* = 0.26) [21]. However, there were low to moderate correlations between vertical jump height and Tegner activity scale, the ACL-RSI scale [34] (*r* = 0.61–0.64), and functional and strength tests (*r* = 0.30–0.57) [21], and these coefficients are slightly higher than the values found in the current study. The differences can be caused by a higher proportion of athletes in the cohort [21], as not all patients in our cohort were involved in organized sports, and patients in the current study were tested 3 months later with substantially more comprehensive correlation analyses.

The current study demonstrated a very low correlation between clinical assessment of laxity with the Rolimeter and functional tests, PROM scores and satisfaction with surgery, and the degree of laxity is therefore not a sufficient outcome in itself. Obviously, it can be used to evaluate technical aspects after ACLR but not function or subjective perception of the result.

The interpretation of Pearson’s rho relies on the validity of the outcomes that are tested. As KOOS, IKDC and the Lysholm score have doubtful content validity [13], it can be expected that any correlation with scores from these PROMs are quite low. However, KNEES-ACL has good content and construct validity [12, 13], and the functional hop tests and Rolimeter measurements have good reliability, so based on the negligible and low correlations demonstrated in the current study it can be concluded that the different measures express various aspects of outcome.

It is a general understanding that PROM scores are the most important outcomes in clinical trials and a valuable basis for the development of treatment strategies. An example of this is the KANON study [7] on acute ACL injuries. It found no difference in KOOS scores between two strategies: ACLR or physiotherapy and optional ACLR, but there was a significant difference in knee stability between the two groups and an insignificant difference in Tegner score and return to sports in favor of ACLR at 2-year follow-up. It was concluded that ACLR was not superior to physiotherapy and optional ACLR, but the results from the current study show that this cannot be characterized as a correct interpretation of the results.

This study relates to correlations one year after ACLR in a mixed group of patients, and correlations may be different in other cohorts, e.g., in elite athletes or at shorter or longer follow-up. There are no longitudinal studies in the literature with sufficient data to describe the changes in the three types of outcomes with time or in different cohorts.

It is a strength of this study that it includes a prospective, consecutive cohort of unselected patients who have been treated with ACLR, that outcome data were produced by independent observers with practical experience in relation to the various tests, and that it uses scores from a PROM with content and construct validity for patients with ACL-injury. It is a limitation in relation to generalize from the results of the study that it is restricted to 1-year follow-up.

Do the results of this study indicate that it is always necessary to include all three types of outcomes in relation to follow-up after ACLR? It depends on the purpose of follow-up. The majority of published manuscripts on the outcome after ACLR are series, and high precision of the outcome is in most cases not essential. In contrast, studies that compare groups require outcomes with high precision, in particular if the aim is to decide which treatment is superior, and it is suggested that all three outcomes are included in such studies. If only one or two are reported, the limitations caused by this should be discussed—a study that only reports PROM scores cannot conclude on stability and function.

## Conclusion

There was no clinically relevant correlation between scores obtained by PROMs, a battery of functional performance tests and instrumented laxity of the knee at 1-year follow-up after ACLR, meaning that the various modalities represent different aspects of outcome and that one type of outcome cannot represent all. This is an argument for always to include and report all three types of outcomes. Conclusions based on one type of outcome may not be sufficient.

### Supplementary Information

Below is the link to the electronic supplementary material.

Supplementary file1 (PDF 597 KB)

## Data Availability

Anonymised data will be available upon reasonable request.
