# Supplementary material for: Functional performance tests, clinical measurements, and patient-reported outcome measures do not correlate as outcomes 1 year after anterior cruciate ligament reconstruction
Source: Knee Surg Sports Traumatol Arthrosc. 2023 Nov 10;31(12):5905–12. doi: 10.1007/s00167-023-07648-w (PMC10719130; doi:10.1007/s00167-023-07648-w)
Supplement: Supplementary file 1 — Supplementary file1 (PDF 597 KB) [file 167_2023_7648_MOESM1_ESM.pdf]

Rolimeter difference injured minus non-injured knee

The MEANS Procedure

Variable	Label	N	N Miss	Mean	Std Dev	Median	25th Pctl	75th Pctl	Minimum	Maximum
_Single_hop_syge_ben_cm	Single hop, injured leg	151	0	100	32	95	75	123	38	184
__6_m_timed_hop_syge_ben	6 m timed hop, injured leg	151	0	13	57	3	2	3	0	419
_Triple_hop_syge_ben	Triple hop, injured leg	151	0	353	99	349	283	416	3	590
_Cross_over_hop_syge_ben	Cross over hop, injured leg	151	0	309	92	300	236	373	87	555
_Gennemsnit___i_forhold_til_rask	Composite symmetry index	151	0	89	12	92	83	97	44	106
Rolimeter	Rolimeter difference injured minus non-injured knee	151	0	1	1	2	1	2	-2	6
Satisfaction	Satisfaction with surgery	151	0	-2	1	-1	-2	-1	-3	-1
Repeat_surgery	Willingness to repeat surgery	151	0	-2	1	-1	-2	-1	-3	0

confidence intervals computed based on Fishers z-transformation

Rolimeter difference injured minus non-injured knee

The CORR Procedure

Pearson Correlation Statistics (Fisher's z Transformation)

Variable	With Variable	N	Sample Correlation	Fisher's z	Bias Adjustment	Correlation Estimate	95% Confidence Limits		p Value for H0:Rho=0
**_Single_hop_syge_ben_cm**	**IKDC**	151	0.29734	0.30660	0.0009911	0.29643	0.143501	0.435541	0.0002
**__6_m_timed_hop_syge_ben**	**IKDC**	151	0.08044	0.08062	0.0002681	0.08018	-0.080584	0.236871	0.3267
**_Triple_hop_syge_ben**	**IKDC**	151	0.32947	0.34223	0.00110	0.32849	0.178102	0.463876	<.0001
**_Cross_over_hop_syge_ben**	**IKDC**	151	0.20650	0.20951	0.0006883	0.20584	0.047681	0.353934	0.0108
**_Gennemsnit___i_forhold_til_rask**	**IKDC**	151	0.38606	0.40716	0.00129	0.38496	0.239992	0.513139	<.0001
**Rolimeter**	**IKDC**	151	0.11039	0.11085	0.0003680	0.11003	-0.050587	0.265100	0.1775
**Satisfaction**	**IKDC**	151	0.53061	0.59099	0.00177	0.52933	0.403741	0.635344	<.0001
**Repeat_surgery**	**IKDC**	151	0.07132	0.07144	0.0002377	0.07108	-0.089667	0.228217	0.3848
**_Single_hop_syge_ben_cm**	**lysholm**	151	0.24352	0.24852	0.0008117	0.24276	0.086380	0.387463	0.0025

Pearson Correlation Statistics (Fisher's z Transformation)

Variable	With Variable	N	Sample Correlation	Fisher's z	Bias Adjustment	Correlation Estimate	95% Confidence Limits		p Value for H0:Rho=0
**__6_m_timed_hop_syge_ben**	**lysholm**	151	0.07801	0.07817	0.0002600	0.07775	-0.083010	0.234565	0.3416
**_Triple_hop_syge_ben**	**lysholm**	151	0.26710	0.27374	0.0008903	0.26627	0.111277	0.408622	0.0009
**_Cross_over_hop_syge_ben**	**lysholm**	151	0.11761	0.11816	0.0003920	0.11722	-0.043318	0.271860	0.1506
**_Gennemsnit___i_forhold_til_rask**	**lysholm**	151	0.35755	0.37408	0.00119	0.35651	0.208669	0.488429	<.0001
**Rolimeter**	**lysholm**	151	0.09538	0.09567	0.0003179	0.09507	-0.065658	0.250985	0.2445
**Satisfaction**	**lysholm**	151	0.42532	0.45417	0.00142	0.42416	0.283652	0.546842	<.0001
**Repeat_surgery**	**lysholm**	151	0.00264	0.00264	8.79465E-6	0.00263	-0.157165	0.162290	0.9744
**_Single_hop_syge_ben_cm**	**Tegner_score**	151	0.45324	0.48878	0.00151	0.45204	0.315064	0.570574	<.0001
**__6_m_timed_hop_syge_ben**	**Tegner_score**	151	-0.01577	-0.01577	-0.0000526	-0.01572	-0.175004	0.144376	0.8479
**_Triple_hop_syge_ben**	**Tegner_score**	151	0.44993	0.48461	0.00150	0.44873	0.311315	0.567764	<.0001
**_Cross_over_hop_syge_ben**	**Tegner_score**	151	0.39163	0.41372	0.00131	0.39052	0.246150	0.517944	<.0001

Pearson Correlation Statistics (Fisher's z Transformation)

Variable	With Variable	N	Sample Correlation	Fisher's z	Bias Adjustment	Correlation Estimate	95% Confidence Limits		p Value for H0:Rho=0
**_Gennemsnit___i_forhold_til_rask**	**Tegner_score**	151	0.37767	0.39734	0.00126	0.37659	0.230739	0.505887	<.0001
**Rolimeter**	**Tegner_score**	151	-0.11085	-0.11130	-0.0003695	-0.11048	-0.265524	0.050132	0.1757
**Satisfaction**	**Tegner_score**	151	0.36879	0.38702	0.00123	0.36773	0.220980	0.498195	<.0001
**Repeat_surgery**	**Tegner_score**	151	-0.00187	-0.00187	-6.221E-6	-0.00186	-0.161541	0.157915	0.9819
**_Single_hop_syge_ben_cm**	**KNEES_ACL_1**	151	0.26531	0.27181	0.0008844	0.26448	0.109376	0.407017	0.0009
**__6_m_timed_hop_syge_ben**	**KNEES_ACL_1**	151	0.02370	0.02370	0.0000790	0.02362	-0.136626	0.182657	0.7731
**_Triple_hop_syge_ben**	**KNEES_ACL_1**	151	0.28756	0.29590	0.0009585	0.28668	0.133041	0.426860	0.0003
**_Cross_over_hop_syge_ben**	**KNEES_ACL_1**	151	0.21379	0.21714	0.0007126	0.21311	0.055266	0.360568	0.0083
**_Gennemsnit___i_forhold_til_rask**	**KNEES_ACL_1**	151	0.32119	0.33297	0.00107	0.32023	0.169153	0.456603	<.0001
**Rolimeter**	**KNEES_ACL_1**	151	0.07898	0.07914	0.0002633	0.07871	-0.082046	0.235481	0.3357
**Satisfaction**	**KNEES_ACL_1**	151	0.52632	0.58504	0.00175	0.52505	0.398767	0.631795	<.0001

Pearson Correlation Statistics (Fisher's z Transformation)

Variable	With Variable	N	Sample Correlation	Fisher's z	Bias Adjustment	Correlation Estimate	95% Confidence Limits		p Value for H0:Rho=0
**Repeat_surgery**	**KNEES_ACL_1**	151	0.09135	0.09161	0.0003045	0.09105	-0.069693	0.247183	0.2651
**_Single_hop_syge_ben_cm**	**KNEES_ACL_2**	151	0.11050	0.11096	0.0003683	0.11014	-0.050477	0.265202	0.1771
**__6_m_timed_hop_syge_ben**	**KNEES_ACL_2**	151	0.03205	0.03206	0.0001068	0.03194	-0.128445	0.190694	0.6965
**_Triple_hop_syge_ben**	**KNEES_ACL_2**	151	0.11963	0.12020	0.0003988	0.11923	-0.041281	0.273748	0.1437
**_Cross_over_hop_syge_ben**	**KNEES_ACL_2**	151	-0.03310	-0.03312	-0.0001103	-0.03299	-0.191712	0.127406	0.6870
**_Gennemsnit___i_forhold_til_rask**	**KNEES_ACL_2**	151	0.10093	0.10127	0.0003364	0.10059	-0.060101	0.256205	0.2179
**Rolimeter**	**KNEES_ACL_2**	151	0.09277	0.09304	0.0003092	0.09247	-0.068269	0.248526	0.2577
**Satisfaction**	**KNEES_ACL_2**	151	0.31539	0.32652	0.00105	0.31445	0.162900	0.451498	<.0001
**Repeat_surgery**	**KNEES_ACL_2**	151	-0.00722	-0.00722	-0.0000241	-0.00720	-0.166734	0.152707	0.9300
**_Single_hop_syge_ben_cm**	**KNEES_ACL_3**	151	0.20794	0.21102	0.0006931	0.20728	0.049176	0.355243	0.0103
**__6_m_timed_hop_syge_ben**	**KNEES_ACL_3**	151	0.03525	0.03526	0.0001175	0.03513	-0.125299	0.193773	0.6679

Pearson Correlation Statistics (Fisher's z Transformation)

Variable	With Variable	N	Sample Correlation	Fisher's z	Bias Adjustment	Correlation Estimate	95% Confidence Limits		p Value for H0:Rho=0
_Triple_hop_syge_ben	KNEES_ACL_3	151	0.22720	0.23124	0.0007573	0.22648	0.069262	0.372729	0.0049
_Cross_over_hop_syge_ben	KNEES_ACL_3	151	0.10988	0.11033	0.0003663	0.10952	-0.051101	0.264621	0.1795
_Gennemsnit___i_forhold_til_rask	KNEES_ACL_3	151	0.26949	0.27632	0.0008983	0.26866	0.113814	0.410760	0.0008
Rolimeter	KNEES_ACL_3	151	0.07177	0.07189	0.0002392	0.07153	-0.089217	0.228647	0.3818
Satisfaction	KNEES_ACL_3	151	0.47557	0.51725	0.00159	0.47434	0.340406	0.589414	<.0001
Repeat_surgery	KNEES_ACL_3	151	0.10205	0.10241	0.0003402	0.10172	-0.058972	0.257264	0.2128
_Single_hop_syge_ben_cm	KNEES_ACL_4	151	0.09799	0.09831	0.0003266	0.09767	-0.063041	0.253446	0.2317
__6_m_timed_hop_syge_ben	KNEES_ACL_4	151	0.02052	0.02053	0.0000684	0.02045	-0.139731	0.179596	0.8028
_Triple_hop_syge_ben	KNEES_ACL_4	151	0.19601	0.19858	0.0006534	0.19539	0.036806	0.344367	0.0157
_Cross_over_hop_syge_ben	KNEES_ACL_4	151	0.05687	0.05693	0.0001896	0.05668	-0.103989	0.214468	0.4886
_Gennemsnit___i_forhold_til_rask	KNEES_ACL_4	151	0.16471	0.16622	0.0005490	0.16417	0.004565	0.315625	0.0432

Pearson Correlation Statistics (Fisher's z Transformation)

Variable	With Variable	N	Sample Correlation	Fisher's z	Bias Adjustment	Correlation Estimate	95% Confidence Limits		p Value for H0:Rho=0
**Rolimeter**	**KNEES_ACL_4**	151	-0.01171	-0.01171	-0.0000390	-0.01167	-0.171076	0.148338	0.8868
**Satisfaction**	**KNEES_ACL_4**	151	0.33046	0.34335	0.00110	0.32948	0.179181	0.464750	<.0001
**Repeat_surgery**	**KNEES_ACL_4**	151	0.08791	0.08814	0.0002930	0.08762	-0.073133	0.243934	0.2836
**_Single_hop_syge_ben_cm**	**KNEES_ACL_5**	151	0.19353	0.19600	0.0006451	0.19291	0.034237	0.342098	0.0171
**__6_m_timed_hop_syge_ben**	**KNEES_ACL_5**	151	0.06772	0.06783	0.0002257	0.06750	-0.093235	0.224804	0.4093
**_Triple_hop_syge_ben**	**KNEES_ACL_5**	151	0.27984	0.28751	0.0009328	0.27898	0.124816	0.419995	0.0005
**_Cross_over_hop_syge_ben**	**KNEES_ACL_5**	151	0.10698	0.10739	0.0003566	0.10663	-0.054019	0.261897	0.1914
**_Gennemsnit___i_forhold_til_rask**	**KNEES_ACL_5**	151	0.24192	0.24681	0.0008064	0.24116	0.084691	0.386016	0.0027
**Rolimeter**	**KNEES_ACL_5**	151	0.18596	0.18815	0.0006199	0.18536	0.026413	0.335165	0.0221
**Satisfaction**	**KNEES_ACL_5**	151	0.38136	0.40165	0.00127	0.38027	0.234807	0.509080	<.0001
**Repeat_surgery**	**KNEES_ACL_5**	151	0.08089	0.08107	0.0002696	0.08062	-0.080140	0.237293	0.3240

Pearson Correlation Statistics (Fisher's z Transformation)

Variable	With Variable	N	Sample Correlation	Fisher's z	Bias Adjustment	Correlation Estimate	95% Confidence Limits		p Value for H0:Rho=0
**_Single_hop_syge_ben_cm**	**KNEES_ACL_6**	151	-0.00160	-0.00160	-5.3285E-6	-0.00159	-0.161281	0.158175	0.9845
**__6_m_timed_hop_syge_ben**	**KNEES_ACL_6**	151	0.01788	0.01788	0.0000596	0.01782	-0.142310	0.177048	0.8278
**_Triple_hop_syge_ben**	**KNEES_ACL_6**	151	0.07124	0.07136	0.0002375	0.07100	-0.089744	0.228144	0.3853
**_Cross_over_hop_syge_ben**	**KNEES_ACL_6**	151	0.0009514	0.0009514	3.17146E-6	0.0009483	-0.158804	0.160652	0.9908
**_Gennemsnit___i_forhold_til_rask**	**KNEES_ACL_6**	151	0.17860	0.18054	0.0005953	0.17803	0.018832	0.328415	0.0281
**Rolimeter**	**KNEES_ACL_6**	151	0.14638	0.14744	0.0004879	0.14591	-0.014152	0.298674	0.0729
**Satisfaction**	**KNEES_ACL_6**	151	0.41028	0.43595	0.00137	0.40914	0.266855	0.533976	<.0001
**Repeat_surgery**	**KNEES_ACL_6**	151	-0.03454	-0.03456	-0.0001151	-0.03443	-0.193096	0.125991	0.6742
**_Single_hop_syge_ben_cm**	**KNEES_ACL_7**	151	0.24052	0.24533	0.0008017	0.23976	0.083223	0.384758	0.0028
**__6_m_timed_hop_syge_ben**	**KNEES_ACL_7**	151	-0.04137	-0.04139	-0.0001379	-0.04123	-0.199643	0.119285	0.6146
**_Triple_hop_syge_ben**	**KNEES_ACL_7**	151	0.25389	0.25957	0.0008463	0.25310	0.097303	0.396786	0.0016

Pearson Correlation Statistics (Fisher's z Transformation)

Variable	With Variable	N	Sample Correlation	Fisher's z	Bias Adjustment	Correlation Estimate	95% Confidence Limits		p Value for H0:Rho=0
**_Cross_over_hop_syge_ben**	**KNEES_ACL_7**	151	0.19182	0.19422	0.0006394	0.19120	0.032463	0.340529	0.0181
**_Gennemsnit___i_forhold_til_rask**	**KNEES_ACL_7**	151	0.36113	0.37819	0.00120	0.36009	0.212584	0.491542	<.0001
**Rolimeter**	**KNEES_ACL_7**	151	-0.02817	-0.02818	-0.0000939	-0.02808	-0.186971	0.132241	0.7317
**Satisfaction**	**KNEES_ACL_7**	151	0.37557	0.39490	0.00125	0.37450	0.228435	0.504075	<.0001
**Repeat_surgery**	**KNEES_ACL_7**	151	-0.06291	-0.06300	-0.0002097	-0.06271	-0.220228	0.098004	0.4434
**_Single_hop_syge_ben_cm**	**KOOS_S**	151	0.01859	0.01859	0.0000620	0.01853	-0.141619	0.177731	0.8211
**__6_m_timed_hop_syge_ben**	**KOOS_S**	151	0.03060	0.03061	0.0001020	0.03050	-0.129862	0.189306	0.7096
**_Triple_hop_syge_ben**	**KOOS_S**	151	0.02428	0.02428	0.0000809	0.02419	-0.136060	0.183215	0.7677
**_Cross_over_hop_syge_ben**	**KOOS_S**	151	-0.05843	-0.05849	-0.0001948	-0.05823	-0.215953	0.102448	0.4767
**_Gennemsnit___i_forhold_til_rask**	**KOOS_S**	151	0.01424	0.01425	0.0000475	0.01420	-0.145862	0.173532	0.8624
**Rolimeter**	**KOOS_S**	151	-0.14828	-0.14938	-0.0004943	-0.14779	-0.300431	0.012222	0.0692

Pearson Correlation Statistics (Fisher's z Transformation)

Variable	With Variable	N	Sample Correlation	Fisher's z	Bias Adjustment	Correlation Estimate	95% Confidence Limits		p Value for H0:Rho=0
**Satisfaction**	**KOOS_S**	151	0.08617	0.08638	0.0002872	0.08588	-0.074873	0.242287	0.2933
**Repeat_surgery**	**KOOS_S**	151	0.07941	0.07958	0.0002647	0.07915	-0.081610	0.235897	0.3330
**_Single_hop_syge_ben_cm**	**KOOS_P**	151	0.24755	0.25280	0.0008252	0.24677	0.090617	0.391087	0.0021
**__6_m_timed_hop_syge_ben**	**KOOS_P**	151	0.06206	0.06214	0.0002069	0.06186	-0.098850	0.219416	0.4497
**_Triple_hop_syge_ben**	**KOOS_P**	151	0.27890	0.28649	0.0009297	0.27804	0.123809	0.419153	0.0005
**_Cross_over_hop_syge_ben**	**KOOS_P**	151	0.18450	0.18663	0.0006150	0.18390	0.024907	0.333826	0.0232
**_Gennemsnit___i_forhold_til_rask**	**KOOS_P**	151	0.26816	0.27488	0.0008939	0.26733	0.112401	0.409569	0.0008
**Rolimeter**	**KOOS_P**	151	0.13529	0.13612	0.0004510	0.13485	-0.025431	0.288363	0.0977
**Satisfaction**	**KOOS_P**	151	0.50082	0.55040	0.00167	0.49956	0.369305	0.610573	<.0001
**Repeat_surgery**	**KOOS_P**	151	0.16045	0.16185	0.0005348	0.15993	0.000203	0.311693	0.0490
**_Single_hop_syge_ben_cm**	**KOOS_A**	151	0.17174	0.17346	0.0005725	0.17119	0.011779	0.322106	0.0348

Pearson Correlation Statistics (Fisher's z Transformation)

Variable	With Variable	N	Sample Correlation	Fisher's z	Bias Adjustment	Correlation Estimate	95% Confidence Limits		p Value for H0:Rho=0
**__6_m_timed_hop_syge_ben**	**KOOS_A**	151	0.03151	0.03152	0.0001050	0.03140	-0.128974	0.190176	0.7014
**_Triple_hop_syge_ben**	**KOOS_A**	151	0.20591	0.20890	0.0006864	0.20526	0.047071	0.353398	0.0110
**_Cross_over_hop_syge_ben**	**KOOS_A**	151	0.10280	0.10317	0.0003427	0.10246	-0.058218	0.257969	0.2095
**_Gennemsnit___i_forhold_til_rask**	**KOOS_A**	151	0.30479	0.31479	0.00102	0.30387	0.151496	0.442139	0.0001
**Rolimeter**	**KOOS_A**	151	0.07005	0.07016	0.0002335	0.06982	-0.090926	0.227013	0.3933
**Satisfaction**	**KOOS_A**	151	0.35416	0.37020	0.00118	0.35313	0.204962	0.485475	<.0001
**Repeat_surgery**	**KOOS_A**	151	0.09196	0.09223	0.0003065	0.09166	-0.069079	0.247762	0.2619
**_Single_hop_syge_ben_cm**	**KOOS_SP**	151	0.30428	0.31423	0.00101	0.30336	0.150944	0.441685	0.0001
**__6_m_timed_hop_syge_ben**	**KOOS_SP**	151	0.01407	0.01407	0.0000469	0.01403	-0.146030	0.173366	0.8641
**_Triple_hop_syge_ben**	**KOOS_SP**	151	0.29723	0.30647	0.0009908	0.29632	0.143381	0.435442	0.0002
**_Cross_over_hop_syge_ben**	**KOOS_SP**	151	0.26316	0.26950	0.0008772	0.26234	0.107101	0.405095	0.0010

Pearson Correlation Statistics (Fisher's z Transformation)

Variable	With Variable	N	Sample Correlation	Fisher's z	Bias Adjustment	Correlation Estimate	95% Confidence Limits		p Value for H0:Rho=0
**_Gennemsnit___i_forhold_til_rask**	**KOOS_SP**	151	0.37404	0.39311	0.00125	0.37297	0.226747	0.502745	<.0001
**Rolimeter**	**KOOS_SP**	151	0.08531	0.08552	0.0002844	0.08503	-0.075727	0.241479	0.2982
**Satisfaction**	**KOOS_SP**	151	0.49835	0.54711	0.00166	0.49710	0.366468	0.608511	<.0001
**Repeat_surgery**	**KOOS_SP**	151	0.10937	0.10981	0.0003646	0.10901	-0.051614	0.264142	0.1816
**_Single_hop_syge_ben_cm**	**KOOS_Q**	151	0.24591	0.25106	0.0008197	0.24514	0.088897	0.389617	0.0023
**__6_m_timed_hop_syge_ben**	**KOOS_Q**	151	0.09466	0.09495	0.0003155	0.09435	-0.066380	0.250305	0.2481
**_Triple_hop_syge_ben**	**KOOS_Q**	151	0.24818	0.25347	0.0008273	0.24740	0.091283	0.391656	0.0020
**_Cross_over_hop_syge_ben**	**KOOS_Q**	151	0.14632	0.14738	0.0004877	0.14584	-0.014217	0.298615	0.0730
**_Gennemsnit___i_forhold_til_rask**	**KOOS_Q**	151	0.34604	0.36094	0.00115	0.34502	0.196101	0.478387	<.0001
**Rolimeter**	**KOOS_Q**	151	0.05657	0.05663	0.0001886	0.05639	-0.104282	0.214185	0.4908
**Satisfaction**	**KOOS_Q**	151	0.49331	0.54043	0.00164	0.49207	0.360687	0.604299	<.0001

Pearson Correlation Statistics (Fisher's z Transformation)

Variable	With Variable	N	Sample Correlation	Fisher's z	Bias Adjustment	Correlation Estimate	95% Confidence Limits		p Value for H0:Rho=0
**Repeat_surgery**	**KOOS_Q**	151	0.10633	0.10674	0.0003544	0.10598	-0.054673	0.261287	0.1941

confidence intervals computed based on Fishers z-transformation

Rolimeter difference injured minus non-injured knee

The CORR Procedure

Pearson Correlation Statistics (Fisher's z Transformation)

Variable	With Variable	N	Sample Correlation	Fisher's z	Bias Adjustment	Correlation Estimate	95% Confidence Limits		p Value for H0:Rho=0
**_Single_hop_syge_ben_cm**	**IKDC**	77	0.30058	0.31016	0.00198	0.29878	0.080169	0.489973	0.0076
**__6_m_timed_hop_syge_ben**	**IKDC**	77	-0.00250	-0.00250	-0.0000164	-0.00248	-0.226336	0.221619	0.9828
**_Triple_hop_syge_ben**	**IKDC**	77	0.29819	0.30754	0.00196	0.29641	0.077577	0.487988	0.0082
**_Cross_over_hop_syge_ben**	**IKDC**	77	0.29025	0.29884	0.00191	0.28850	0.068977	0.481372	0.0101

Pearson Correlation Statistics (Fisher's z Transformation)

Variable	With Variable	N	Sample Correlation	Fisher's z	Bias Adjustment	Correlation Estimate	95% Confidence Limits		p Value for H0:Rho=0
**_Gennemsnit___i_forhold_til_rask**	**IKDC**	77	0.31400	0.32498	0.00207	0.31214	0.094786	0.501085	0.0052
**Rolimeter**	**IKDC**	77	0.13540	0.13624	0.0008908	0.13453	-0.092229	0.348022	0.2412
**Satisfaction**	**IKDC**	77	0.56686	0.64288	0.00373	0.56432	0.389582	0.699841	<.0001
**Repeat_surgery**	**IKDC**	77	0.02680	0.02680	0.0001763	0.02662	-0.198542	0.249115	0.8176
**_Single_hop_syge_ben_cm**	**lysholm**	77	0.27125	0.27821	0.00178	0.26959	0.048544	0.465464	0.0167
**__6_m_timed_hop_syge_ben**	**lysholm**	77	0.08052	0.08069	0.0005297	0.07999	-0.146616	0.298619	0.4876
**_Triple_hop_syge_ben**	**lysholm**	77	0.24818	0.25347	0.00163	0.24664	0.023990	0.445985	0.0292
**_Cross_over_hop_syge_ben**	**lysholm**	77	0.22295	0.22676	0.00147	0.22156	-0.002546	0.424474	0.0511
**_Gennemsnit___i_forhold_til_rask**	**lysholm**	77	0.22137	0.22510	0.00146	0.21999	-0.004198	0.423119	0.0528
**Rolimeter**	**lysholm**	77	0.07168	0.07181	0.0004716	0.07121	-0.155242	0.290557	0.5368
**Satisfaction**	**lysholm**	77	0.37387	0.39291	0.00246	0.37175	0.161193	0.549939	0.0007

Pearson Correlation Statistics (Fisher's z Transformation)

Variable	With Variable	N	Sample Correlation	Fisher's z	Bias Adjustment	Correlation Estimate	95% Confidence Limits		p Value for H0:Rho=0
**Repeat_surgery**	**lysholm**	77	-0.13243	-0.13321	-0.0008713	-0.13158	-0.345376	0.095210	0.2518
**_Single_hop_syge_ben_cm**	**Tegner_score**	77	0.38454	0.40538	0.00253	0.38239	0.173244	0.558529	0.0005
**__6_m_timed_hop_syge_ben**	**Tegner_score**	77	-0.07022	-0.07034	-0.0004620	-0.06976	-0.289223	0.156663	0.5451
**_Triple_hop_syge_ben**	**Tegner_score**	77	0.41852	0.44589	0.00275	0.41624	0.212034	0.585626	0.0001
**_Cross_over_hop_syge_ben**	**Tegner_score**	77	0.41875	0.44618	0.00275	0.41648	0.212304	0.585812	0.0001
**_Gennemsnit___i_forhold_til_rask**	**Tegner_score**	77	0.37563	0.39496	0.00247	0.37350	0.163176	0.551358	0.0007
**Rolimeter**	**Tegner_score**	77	-0.15620	-0.15749	-0.00103	-0.15519	-0.366435	0.071262	0.1755
**Satisfaction**	**Tegner_score**	77	0.41337	0.43967	0.00272	0.41112	0.206117	0.581546	0.0002
**Repeat_surgery**	**Tegner_score**	77	-0.03615	-0.03616	-0.0002378	-0.03591	-0.257815	0.189594	0.7557
**_Single_hop_syge_ben_cm**	**KNEES_ACL_1**	77	0.30873	0.31914	0.00203	0.30689	0.089033	0.496727	0.0060
**__6_m_timed_hop_syge_ben**	**KNEES_ACL_1**	77	-0.07098	-0.07110	-0.0004670	-0.07051	-0.289915	0.155927	0.5408

Pearson Correlation Statistics (Fisher's z Transformation)

Variable	With Variable	N	Sample Correlation	Fisher's z	Bias Adjustment	Correlation Estimate	95% Confidence Limits		p Value for H0:Rho=0
_Triple_hop_syge_ben	KNEES_ACL_1	77	0.32688	0.33934	0.00215	0.32496	0.108911	0.511698	0.0035
_Cross_over_hop_syge_ben	KNEES_ACL_1	77	0.34178	0.35611	0.00225	0.33979	0.125353	0.523899	0.0022
_Gennemsnit___i_forhold_til_rask	KNEES_ACL_1	77	0.26852	0.27527	0.00177	0.26688	0.045632	0.463175	0.0179
Rolimeter	KNEES_ACL_1	77	0.03313	0.03314	0.0002179	0.03291	-0.192489	0.255008	0.7756
Satisfaction	KNEES_ACL_1	77	0.46514	0.50385	0.00306	0.46274	0.266369	0.622229	<.0001
Repeat_surgery	KNEES_ACL_1	77	0.04881	0.04885	0.0003211	0.04849	-0.177413	0.269544	0.6743
_Single_hop_syge_ben_cm	KNEES_ACL_2	77	0.24189	0.24678	0.00159	0.24039	0.017350	0.440647	0.0338
__6_m_timed_hop_syge_ben	KNEES_ACL_2	77	-0.09747	-0.09779	-0.0006413	-0.09684	-0.314008	0.129958	0.4002
_Triple_hop_syge_ben	KNEES_ACL_2	77	0.19831	0.20097	0.00130	0.19706	-0.028165	0.403238	0.0838
_Cross_over_hop_syge_ben	KNEES_ACL_2	77	0.15054	0.15169	0.0009904	0.14957	-0.076984	0.361444	0.1919
_Gennemsnit___i_forhold_til_rask	KNEES_ACL_2	77	0.16390	0.16539	0.00108	0.16285	-0.063442	0.373217	0.1548

Pearson Correlation Statistics (Fisher's z Transformation)

Variable	With Variable	N	Sample Correlation	Fisher's z	Bias Adjustment	Correlation Estimate	95% Confidence Limits		p Value for H0:Rho=0
**Rolimeter**	**KNEES_ACL_2**	77	0.06117	0.06125	0.0004024	0.06077	-0.165461	0.280925	0.5983
**Satisfaction**	**KNEES_ACL_2**	77	0.25515	0.26091	0.00168	0.25358	0.031380	0.451890	0.0248
**Repeat_surgery**	**KNEES_ACL_2**	77	-0.10753	-0.10795	-0.0007074	-0.10683	-0.323077	0.120022	0.3531
**_Single_hop_syge_ben_cm**	**KNEES_ACL_3**	77	0.29374	0.30266	0.00193	0.29197	0.072753	0.484283	0.0092
**__6_m_timed_hop_syge_ben**	**KNEES_ACL_3**	77	0.00618	0.00618	0.0000407	0.00614	-0.218136	0.229806	0.9576
**_Triple_hop_syge_ben**	**KNEES_ACL_3**	77	0.24205	0.24696	0.00159	0.24055	0.017520	0.440784	0.0336
**_Cross_over_hop_syge_ben**	**KNEES_ACL_3**	77	0.24823	0.25353	0.00163	0.24670	0.024046	0.446030	0.0292
**_Gennemsnit___i_forhold_til_rask**	**KNEES_ACL_3**	77	0.23845	0.24313	0.00157	0.23697	0.013715	0.437713	0.0365
**Rolimeter**	**KNEES_ACL_3**	77	0.09009	0.09034	0.0005927	0.08950	-0.137225	0.307322	0.4371
**Satisfaction**	**KNEES_ACL_3**	77	0.41606	0.44292	0.00274	0.41379	0.209204	0.583677	0.0001
**Repeat_surgery**	**KNEES_ACL_3**	77	0.02269	0.02269	0.0001492	0.02254	-0.202465	0.245277	0.8453

Pearson Correlation Statistics (Fisher's z Transformation)

Variable	With Variable	N	Sample Correlation	Fisher's z	Bias Adjustment	Correlation Estimate	95% Confidence Limits		p Value for H0:Rho=0
**_Single_hop_syge_ben_cm**	**KNEES_ACL_4**	77	0.05273	0.05278	0.0003469	0.05239	-0.173630	0.273160	0.6498
**__6_m_timed_hop_syge_ben**	**KNEES_ACL_4**	77	-0.03911	-0.03913	-0.0002573	-0.03885	-0.260561	0.186755	0.7364
**_Triple_hop_syge_ben**	**KNEES_ACL_4**	77	0.08926	0.08949	0.0005872	0.08867	-0.138048	0.306563	0.4414
**_Cross_over_hop_syge_ben**	**KNEES_ACL_4**	77	0.05218	0.05223	0.0003433	0.05184	-0.174163	0.272651	0.6532
**_Gennemsnit___i_forhold_til_rask**	**KNEES_ACL_4**	77	0.13043	0.13118	0.0008581	0.12959	-0.097210	0.343596	0.2591
**Rolimeter**	**KNEES_ACL_4**	77	-0.02658	-0.02658	-0.0001748	-0.02640	-0.248907	0.198754	0.8191
**Satisfaction**	**KNEES_ACL_4**	77	0.29847	0.30784	0.00196	0.29668	0.077879	0.488219	0.0081
**Repeat_surgery**	**KNEES_ACL_4**	77	-0.0001142	-0.0001142	-7.5139E-7	-0.0001135	-0.224087	0.223871	0.9992
**_Single_hop_syge_ben_cm**	**KNEES_ACL_5**	77	0.11163	0.11210	0.0007344	0.11091	-0.115952	0.326769	0.3349
**__6_m_timed_hop_syge_ben**	**KNEES_ACL_5**	77	0.00221	0.00221	0.0000145	0.00220	-0.221892	0.226064	0.9848

Pearson Correlation Statistics (Fisher's z Transformation)

Variable	With Variable	N	Sample Correlation	Fisher's z	Bias Adjustment	Correlation Estimate	95% Confidence Limits		p Value for H0:Rho=0
**_Triple_hop_syge_ben**	**KNEES_ACL_5**	77	0.11777	0.11832	0.0007748	0.11700	-0.109854	0.332274	0.3088
**_Cross_over_hop_syge_ben**	**KNEES_ACL_5**	77	0.05572	0.05578	0.0003666	0.05536	-0.170737	0.275917	0.6313
**_Gennemsnit___i_forhold_til_rask**	**KNEES_ACL_5**	77	0.30334	0.31320	0.00200	0.30153	0.083167	0.492263	0.0071
**Rolimeter**	**KNEES_ACL_5**	77	0.09485	0.09513	0.0006240	0.09423	-0.132548	0.311630	0.4132
**Satisfaction**	**KNEES_ACL_5**	77	0.36750	0.38553	0.00242	0.36540	0.154032	0.544795	0.0009
**Repeat_surgery**	**KNEES_ACL_5**	77	0.09477	0.09505	0.0006235	0.09415	-0.132628	0.311557	0.4136
**_Single_hop_syge_ben_cm**	**KNEES_ACL_6**	77	-0.12299	-0.12362	-0.0008091	-0.12219	-0.336951	0.104649	0.2876
**__6_m_timed_hop_syge_ben**	**KNEES_ACL_6**	77	-0.11417	-0.11467	-0.0007511	-0.11343	-0.329049	0.113431	0.3239
**_Triple_hop_syge_ben**	**KNEES_ACL_6**	77	-0.03461	-0.03462	-0.0002277	-0.03438	-0.256384	0.191070	0.7658
**_Cross_over_hop_syge_ben**	**KNEES_ACL_6**	77	-0.05121	-0.05125	-0.0003369	-0.05087	-0.271756	0.175100	0.6593
**_Gennemsnit___i_forhold_til_rask**	**KNEES_ACL_6**	77	0.14600	0.14705	0.0009605	0.14506	-0.081572	0.357424	0.2059

Pearson Correlation Statistics (Fisher's z Transformation)

Variable	With Variable	N	Sample Correlation	Fisher's z	Bias Adjustment	Correlation Estimate	95% Confidence Limits		p Value for H0:Rho=0
**Rolimeter**	**KNEES_ACL_6**	77	0.10641	0.10681	0.0007000	0.10571	-0.121134	0.322066	0.3582
**Satisfaction**	**KNEES_ACL_6**	77	0.30439	0.31435	0.00200	0.30257	0.084308	0.493132	0.0068
**Repeat_surgery**	**KNEES_ACL_6**	77	-0.10571	-0.10611	-0.0006955	-0.10502	-0.321441	0.121821	0.3614
**_Single_hop_syge_ben_cm**	**KNEES_ACL_7**	77	0.27880	0.28638	0.00183	0.27711	0.056646	0.471805	0.0138
**__6_m_timed_hop_syge_ben**	**KNEES_ACL_7**	77	-0.13651	-0.13736	-0.0008981	-0.13562	-0.349002	0.091122	0.2373
**_Triple_hop_syge_ben**	**KNEES_ACL_7**	77	0.24556	0.25068	0.00162	0.24404	0.021222	0.443764	0.0310
**_Cross_over_hop_syge_ben**	**KNEES_ACL_7**	77	0.21298	0.21629	0.00140	0.21164	-0.012952	0.415904	0.0628
**_Gennemsnit___i_forhold_til_rask**	**KNEES_ACL_7**	77	0.25809	0.26406	0.00170	0.25650	0.034508	0.454378	0.0231
**Rolimeter**	**KNEES_ACL_7**	77	-0.12109	-0.12169	-0.0007967	-0.12031	-0.335253	0.106542	0.2952
**Satisfaction**	**KNEES_ACL_7**	77	0.37531	0.39459	0.00247	0.37318	0.162813	0.551099	0.0007
**Repeat_surgery**	**KNEES_ACL_7**	77	-0.02516	-0.02517	-0.0001655	-0.02500	-0.247589	0.200103	0.8286

Pearson Correlation Statistics (Fisher's z Transformation)

Variable	With Variable	N	Sample Correlation	Fisher's z	Bias Adjustment	Correlation Estimate	95% Confidence Limits		p Value for H0:Rho=0
**_Single_hop_syge_ben_cm**	**KOOS_S**	77	0.00810	0.00810	0.0000533	0.00805	-0.216322	0.231609	0.9444
**__6_m_timed_hop_syge_ben**	**KOOS_S**	77	0.12017	0.12075	0.0007906	0.11939	-0.107464	0.334425	0.2989
**_Triple_hop_syge_ben**	**KOOS_S**	77	0.02588	0.02589	0.0001703	0.02571	-0.199417	0.248260	0.8238
**_Cross_over_hop_syge_ben**	**KOOS_S**	77	-0.00273	-0.00273	-0.0000179	-0.00271	-0.226549	0.221406	0.9813
**_Gennemsnit___i_forhold_til_rask**	**KOOS_S**	77	0.11886	0.11942	0.0007820	0.11809	-0.108766	0.333254	0.3043
**Rolimeter**	**KOOS_S**	77	-0.26982	-0.27666	-0.00178	-0.26817	-0.464262	-0.047014	0.0173
**Satisfaction**	**KOOS_S**	77	0.04403	0.04406	0.0002897	0.04374	-0.182018	0.265126	0.7047
**Repeat_surgery**	**KOOS_S**	77	0.05349	0.05354	0.0003519	0.05314	-0.172895	0.273861	0.6451
**_Single_hop_syge_ben_cm**	**KOOS_P**	77	0.29665	0.30585	0.00195	0.29487	0.075907	0.486707	0.0085
**__6_m_timed_hop_syge_ben**	**KOOS_P**	77	-0.00358	-0.00358	-0.0000236	-0.00356	-0.227355	0.220597	0.9754
**_Triple_hop_syge_ben**	**KOOS_P**	77	0.26375	0.27013	0.00174	0.26213	0.040536	0.459155	0.0201

Pearson Correlation Statistics (Fisher's z Transformation)

Variable	With Variable	N	Sample Correlation	Fisher's z	Bias Adjustment	Correlation Estimate	95% Confidence Limits		p Value for H0:Rho=0
**_Cross_over_hop_syge_ben**	**KOOS_P**	77	0.30802	0.31836	0.00203	0.30619	0.088263	0.496142	0.0062
**_Gennemsnit___i_forhold_til_rask**	**KOOS_P**	77	0.12438	0.12503	0.0008183	0.12358	-0.103262	0.338194	0.2821
**Rolimeter**	**KOOS_P**	77	0.14436	0.14538	0.0009498	0.14343	-0.083219	0.355976	0.2111
**Satisfaction**	**KOOS_P**	77	0.44248	0.47531	0.00291	0.44013	0.239792	0.604517	<.0001
**Repeat_surgery**	**KOOS_P**	77	0.14922	0.15034	0.0009817	0.14826	-0.078319	0.360276	0.1959
**_Single_hop_syge_ben_cm**	**KOOS_A**	77	0.13698	0.13785	0.0009012	0.13610	-0.090645	0.349424	0.2357
**__6_m_timed_hop_syge_ben**	**KOOS_A**	77	-0.04072	-0.04074	-0.0002679	-0.04045	-0.262055	0.185207	0.7260
**_Triple_hop_syge_ben**	**KOOS_A**	77	0.14065	0.14159	0.0009253	0.13974	-0.086961	0.352680	0.2232
**_Cross_over_hop_syge_ben**	**KOOS_A**	77	0.16715	0.16873	0.00110	0.16608	-0.060138	0.376068	0.1466
**_Gennemsnit___i_forhold_til_rask**	**KOOS_A**	77	0.14418	0.14520	0.0009486	0.14325	-0.083400	0.355817	0.2117
**Rolimeter**	**KOOS_A**	77	0.07370	0.07384	0.0004849	0.07322	-0.153272	0.292404	0.5253

Pearson Correlation Statistics (Fisher's z Transformation)

Variable	With Variable	N	Sample Correlation	Fisher's z	Bias Adjustment	Correlation Estimate	95% Confidence Limits		p Value for H0:Rho=0
**Satisfaction**	**KOOS_A**	77	0.24357	0.24857	0.00160	0.24206	0.019123	0.442076	0.0325
**Repeat_surgery**	**KOOS_A**	77	0.07897	0.07913	0.0005195	0.07845	-0.148133	0.297206	0.4961
**_Single_hop_syge_ben_cm**	**KOOS_SP**	77	0.34814	0.36333	0.00229	0.34613	0.132417	0.529091	0.0018
**__6_m_timed_hop_syge_ben**	**KOOS_SP**	77	-0.12008	-0.12066	-0.0007900	-0.11930	-0.334348	0.107550	0.2993
**_Triple_hop_syge_ben**	**KOOS_SP**	77	0.34439	0.35906	0.00227	0.34239	0.128245	0.526028	0.0020
**_Cross_over_hop_syge_ben**	**KOOS_SP**	77	0.35728	0.37376	0.00235	0.35523	0.142595	0.536520	0.0013
**_Gennemsnit___i_forhold_til_rask**	**KOOS_SP**	77	0.32676	0.33920	0.00215	0.32484	0.108773	0.511594	0.0035
**Rolimeter**	**KOOS_SP**	77	0.05661	0.05667	0.0003724	0.05624	-0.169882	0.276730	0.6259
**Satisfaction**	**KOOS_SP**	77	0.51514	0.56971	0.00339	0.51265	0.326117	0.660759	<.0001
**Repeat_surgery**	**KOOS_SP**	77	0.10417	0.10455	0.0006853	0.10349	-0.123346	0.320052	0.3685
**_Single_hop_syge_ben_cm**	**KOOS_Q**	77	0.22203	0.22579	0.00146	0.22064	-0.003516	0.423678	0.0521

Pearson Correlation Statistics (Fisher's z Transformation)

Variable	With Variable	N	Sample Correlation	Fisher's z	Bias Adjustment	Correlation Estimate	95% Confidence Limits		p Value for H0:Rho=0
**__6_m_timed_hop_syge_ben**	**KOOS_Q**	77	0.01772	0.01772	0.0001166	0.01760	-0.207194	0.240632	0.8788
**_Triple_hop_syge_ben**	**KOOS_Q**	77	0.19044	0.19279	0.00125	0.18923	-0.036289	0.396406	0.0972
**_Cross_over_hop_syge_ben**	**KOOS_Q**	77	0.14583	0.14688	0.0009594	0.14490	-0.081736	0.357279	0.2064
**_Gennemsnit___i_forhold_til_rask**	**KOOS_Q**	77	0.27854	0.28609	0.00183	0.27684	0.056360	0.471582	0.0139
**Rolimeter**	**KOOS_Q**	77	0.01589	0.01589	0.0001045	0.01578	-0.208936	0.238916	0.8913
**Satisfaction**	**KOOS_Q**	77	0.44098	0.47345	0.00290	0.43864	0.238051	0.603344	<.0001
**Repeat_surgery**	**KOOS_Q**	77	0.10378	0.10415	0.0006827	0.10310	-0.123735	0.319697	0.3703

confidence intervals computed based on Fishers z-transformation

---

Rolimeter difference injured minus non-injured knee

The CORR Procedure

Pearson Correlation Statistics (Fisher's z Transformation)

Variable	With Variable	N	Sample Correlation	Fisher's z	Bias Adjustment	Correlation Estimate	95% Confidence Limits		p Value for H0:Rho=0
**_Single_hop_syge_ben_cm**	**IKDC**	74	0.30787	0.31819	0.00211	0.30596	0.083282	0.499534	0.0073
**__6_m_timed_hop_syge_ben**	**IKDC**	74	0.17680	0.17868	0.00121	0.17563	-0.055084	0.388533	0.1322
**_Triple_hop_syge_ben**	**IKDC**	74	0.35562	0.37186	0.00244	0.35349	0.135969	0.538490	0.0017
**_Cross_over_hop_syge_ben**	**IKDC**	74	0.14051	0.14144	0.0009624	0.13956	-0.091864	0.356688	0.2333
**_Gennemsnit___i_forhold_til_rask**	**IKDC**	74	0.45188	0.48706	0.00310	0.44941	0.246196	0.614780	<.0001
**Rolimeter**	**IKDC**	74	0.08257	0.08276	0.0005656	0.08201	-0.149283	0.304800	0.4856
**Satisfaction**	**IKDC**	74	0.49599	0.54397	0.00340	0.49342	0.298591	0.648776	<.0001
**Repeat_surgery**	**IKDC**	74	0.10931	0.10975	0.0007487	0.10857	-0.122978	0.328910	0.3551
**_Single_hop_syge_ben_cm**	**lysholm**	74	0.22055	0.22424	0.00151	0.21912	-0.009878	0.426272	0.0588
**__6_m_timed_hop_syge_ben**	**lysholm**	74	0.07570	0.07584	0.0005185	0.07518	-0.155995	0.298553	0.5228
**_Triple_hop_syge_ben**	**lysholm**	74	0.28260	0.29051	0.00194	0.28082	0.055906	0.478606	0.0144

Pearson Correlation Statistics (Fisher's z Transformation)

Variable	With Variable	N	Sample Correlation	Fisher's z	Bias Adjustment	Correlation Estimate	95% Confidence Limits		p Value for H0:Rho=0
**_Cross_over_hop_syge_ben**	**lysholm**	74	0.03047	0.03048	0.0002087	0.03026	-0.199615	0.256986	0.7973
**_Gennemsnit___i_forhold_til_rask**	**lysholm**	74	0.47567	0.51737	0.00326	0.47314	0.274296	0.633184	<.0001
**Rolimeter**	**lysholm**	74	0.11544	0.11596	0.0007907	0.11466	-0.116901	0.334398	0.3285
**Satisfaction**	**lysholm**	74	0.46640	0.50546	0.00319	0.46390	0.263309	0.626036	<.0001
**Repeat_surgery**	**lysholm**	74	0.12152	0.12213	0.0008324	0.12070	-0.110853	0.339829	0.3035
**_Single_hop_syge_ben_cm**	**Tegner_score**	74	0.53494	0.59704	0.00366	0.53232	0.345896	0.678314	<.0001
**__6_m_timed_hop_syge_ben**	**Tegner_score**	74	0.05800	0.05807	0.0003973	0.05761	-0.173171	0.282389	0.6246
**_Triple_hop_syge_ben**	**Tegner_score**	74	0.48996	0.53601	0.00336	0.48741	0.291360	0.644166	<.0001
**_Cross_over_hop_syge_ben**	**Tegner_score**	74	0.37036	0.38885	0.00254	0.36817	0.152505	0.550372	0.0011
**_Gennemsnit___i_forhold_til_rask**	**Tegner_score**	74	0.38482	0.40570	0.00264	0.38257	0.168829	0.561945	0.0006

Pearson Correlation Statistics (Fisher's z Transformation)

Variable	With Variable	N	Sample Correlation	Fisher's z	Bias Adjustment	Correlation Estimate	95% Confidence Limits		p Value for H0:Rho=0
**Rolimeter**	**Tegner_score**	74	-0.05968	-0.05975	-0.0004088	-0.05927	-0.283924	0.171551	0.6146
**Satisfaction**	**Tegner_score**	74	0.35331	0.36922	0.00242	0.35119	0.133399	0.536629	0.0019
**Repeat_surgery**	**Tegner_score**	74	0.03447	0.03448	0.0002361	0.03423	-0.195800	0.260690	0.7714
**_Single_hop_syge_ben_cm**	**KNEES_ACL_1**	74	0.22918	0.23332	0.00157	0.22769	-0.000853	0.433629	0.0493
**__6_m_timed_hop_syge_ben**	**KNEES_ACL_1**	74	0.12187	0.12248	0.0008347	0.12105	-0.110507	0.340139	0.3021
**_Triple_hop_syge_ben**	**KNEES_ACL_1**	74	0.25813	0.26410	0.00177	0.25648	0.029723	0.458130	0.0261
**_Cross_over_hop_syge_ben**	**KNEES_ACL_1**	74	0.11793	0.11848	0.0008077	0.11713	-0.114427	0.336623	0.3181
**_Gennemsnit___i_forhold_til_rask**	**KNEES_ACL_1**	74	0.36152	0.37863	0.00248	0.35936	0.142571	0.543253	0.0014
**Rolimeter**	**KNEES_ACL_1**	74	0.11751	0.11806	0.0008049	0.11672	-0.114843	0.336249	0.3198
**Satisfaction**	**KNEES_ACL_1**	74	0.57717	0.65821	0.00395	0.57453	0.398321	0.709840	<.0001

Pearson Correlation Statistics (Fisher's z Transformation)

Variable	With Variable	N	Sample Correlation	Fisher's z	Bias Adjustment	Correlation Estimate	95% Confidence Limits		p Value for H0:Rho=0
**Repeat_surgery**	**KNEES_ACL_1**	74	0.12504	0.12570	0.0008565	0.12420	-0.107345	0.342965	0.2895
**_Single_hop_syge_ben_cm**	**KNEES_ACL_2**	74	-0.00757	-0.00757	-0.0000518	-0.00751	-0.235609	0.221364	0.9492
**__6_m_timed_hop_syge_ben**	**KNEES_ACL_2**	74	0.16089	0.16230	0.00110	0.15982	-0.071285	0.374635	0.1714
**_Triple_hop_syge_ben**	**KNEES_ACL_2**	74	0.06449	0.06458	0.0004417	0.06405	-0.166889	0.288331	0.5863
**_Cross_over_hop_syge_ben**	**KNEES_ACL_2**	74	-0.16530	-0.16683	-0.00113	-0.16419	-0.378492	0.066812	0.1598
**_Gennemsnit___i_forhold_til_rask**	**KNEES_ACL_2**	74	0.05506	0.05511	0.0003771	0.05468	-0.176018	0.279685	0.6424
**Rolimeter**	**KNEES_ACL_2**	74	0.12031	0.12089	0.0008240	0.11949	-0.112066	0.338743	0.3084
**Satisfaction**	**KNEES_ACL_2**	74	0.36691	0.38485	0.00251	0.36474	0.148625	0.547598	0.0012
**Repeat_surgery**	**KNEES_ACL_2**	74	0.06892	0.06903	0.0004720	0.06845	-0.162593	0.292374	0.5608
**_Single_hop_syge_ben_cm**	**KNEES_ACL_3**	74	0.12926	0.12999	0.0008853	0.12839	-0.103136	0.346716	0.2734

Pearson Correlation Statistics (Fisher's z Transformation)

Variable	With Variable	N	Sample Correlation	Fisher's z	Bias Adjustment	Correlation Estimate	95% Confidence Limits		p Value for H0:Rho=0
**__6_m_timed_hop_syge_ben**	**KNEES_ACL_3**	74	0.07386	0.07399	0.0005059	0.07336	-0.157788	0.296878	0.5330
**_Triple_hop_syge_ben**	**KNEES_ACL_3**	74	0.21634	0.21981	0.00148	0.21493	-0.014274	0.422667	0.0640
**_Cross_over_hop_syge_ben**	**KNEES_ACL_3**	74	-0.01786	-0.01786	-0.0001223	-0.01774	-0.245244	0.211617	0.8804
**_Gennemsnit___i_forhold_til_rask**	**KNEES_ACL_3**	74	0.31624	0.32746	0.00217	0.31429	0.092427	0.506419	0.0058
**Rolimeter**	**KNEES_ACL_3**	74	0.04229	0.04232	0.0002897	0.04201	-0.188301	0.267933	0.7214
**Satisfaction**	**KNEES_ACL_3**	74	0.52171	0.57869	0.00357	0.51911	0.329720	0.668333	<.0001
**Repeat_surgery**	**KNEES_ACL_3**	74	0.18625	0.18845	0.00128	0.18502	-0.045398	0.396746	0.1123
**_Single_hop_syge_ben_cm**	**KNEES_ACL_4**	74	0.14836	0.14947	0.00102	0.14737	-0.083958	0.363622	0.2079
**__6_m_timed_hop_syge_ben**	**KNEES_ACL_4**	74	0.08931	0.08955	0.0006117	0.08870	-0.142686	0.310902	0.4505
**_Triple_hop_syge_ben**	**KNEES_ACL_4**	74	0.28568	0.29385	0.00196	0.28388	0.059224	0.481168	0.0133

Pearson Correlation Statistics (Fisher's z Transformation)

Variable	With Variable	N	Sample Correlation	Fisher's z	Bias Adjustment	Correlation Estimate	95% Confidence Limits		p Value for H0:Rho=0
**_Cross_over_hop_syge_ben**	**KNEES_ACL_4**	74	0.06148	0.06156	0.0004211	0.06106	-0.169805	0.285577	0.6040
**_Gennemsnit___i_forhold_til_rask**	**KNEES_ACL_4**	74	0.19443	0.19694	0.00133	0.19315	-0.036978	0.403829	0.0970
**Rolimeter**	**KNEES_ACL_4**	74	0.00105	0.00105	7.18803E-6	0.00104	-0.227511	0.229486	0.9929
**Satisfaction**	**KNEES_ACL_4**	74	0.35833	0.37497	0.00245	0.35619	0.139005	0.540683	0.0016
**Repeat_surgery**	**KNEES_ACL_4**	74	0.16540	0.16693	0.00113	0.16429	-0.066709	0.378580	0.1596
**_Single_hop_syge_ben_cm**	**KNEES_ACL_5**	74	0.28180	0.28964	0.00193	0.28002	0.055045	0.477940	0.0147
**__6_m_timed_hop_syge_ben**	**KNEES_ACL_5**	74	0.13881	0.13971	0.0009507	0.13787	-0.093574	0.355182	0.2391
**_Triple_hop_syge_ben**	**KNEES_ACL_5**	74	0.40518	0.42983	0.00278	0.40285	0.192033	0.578135	0.0003
**_Cross_over_hop_syge_ben**	**KNEES_ACL_5**	74	0.14857	0.14968	0.00102	0.14757	-0.083748	0.363805	0.2072
**_Gennemsnit___i_forhold_til_rask**	**KNEES_ACL_5**	74	0.19421	0.19671	0.00133	0.19293	-0.037211	0.403633	0.0974
**Rolimeter**	**KNEES_ACL_5**	74	0.26276	0.26907	0.00180	0.26108	0.034648	0.462016	0.0234

Pearson Correlation Statistics (Fisher's z Transformation)

Variable	With Variable	N	Sample Correlation	Fisher's z	Bias Adjustment	Correlation Estimate	95% Confidence Limits		p Value for H0:Rho=0
**Satisfaction**	**KNEES_ACL_5**	74	0.38708	0.40836	0.00265	0.38482	0.171392	0.563748	0.0006
**Repeat_surgery**	**KNEES_ACL_5**	74	0.06861	0.06872	0.0004699	0.06814	-0.162891	0.292094	0.5626
**_Single_hop_syge_ben_cm**	**KNEES_ACL_6**	74	0.14941	0.15053	0.00102	0.14840	-0.082905	0.364542	0.2047
**__6_m_timed_hop_syge_ben**	**KNEES_ACL_6**	74	0.18386	0.18597	0.00126	0.18264	-0.047857	0.394667	0.1171
**_Triple_hop_syge_ben**	**KNEES_ACL_6**	74	0.16646	0.16802	0.00114	0.16535	-0.065628	0.379510	0.1568
**_Cross_over_hop_syge_ben**	**KNEES_ACL_6**	74	0.05198	0.05202	0.0003560	0.05162	-0.178990	0.276853	0.6611
**_Gennemsnit___i_forhold_til_rask**	**KNEES_ACL_6**	74	0.21285	0.21616	0.00146	0.21146	-0.017904	0.419680	0.0686
**Rolimeter**	**KNEES_ACL_6**	74	0.18298	0.18507	0.00125	0.18177	-0.048754	0.393907	0.1189
**Satisfaction**	**KNEES_ACL_6**	74	0.49462	0.54216	0.00339	0.49206	0.296946	0.647729	<.0001
**Repeat_surgery**	**KNEES_ACL_6**	74	0.03176	0.03177	0.0002175	0.03154	-0.198389	0.258178	0.7889
**_Single_hop_syge_ben_cm**	**KNEES_ACL_7**	74	0.20262	0.20546	0.00139	0.20129	-0.028522	0.410889	0.0834

Pearson Correlation Statistics (Fisher's z Transformation)

Variable	With Variable	N	Sample Correlation	Fisher's z	Bias Adjustment	Correlation Estimate	95% Confidence Limits		p Value for H0:Rho=0
**__6_m_timed_hop_syge_ben**	**KNEES_ACL_7**	74	0.06119	0.06127	0.0004191	0.06078	-0.170085	0.285313	0.6057
**_Triple_hop_syge_ben**	**KNEES_ACL_7**	74	0.26198	0.26824	0.00179	0.26031	0.033824	0.461367	0.0238
**_Cross_over_hop_syge_ben**	**KNEES_ACL_7**	74	0.17449	0.17629	0.00120	0.17333	-0.057448	0.386517	0.1374
**_Gennemsnit___i_forhold_til_rask**	**KNEES_ACL_7**	74	0.44395	0.47714	0.00304	0.44151	0.236911	0.608609	<.0001
**Rolimeter**	**KNEES_ACL_7**	74	0.05501	0.05507	0.0003768	0.05463	-0.176062	0.279642	0.6427
**Satisfaction**	**KNEES_ACL_7**	74	0.38938	0.41106	0.00267	0.38711	0.174004	0.565582	0.0005
**Repeat_surgery**	**KNEES_ACL_7**	74	-0.09364	-0.09391	-0.0006414	-0.09300	-0.314811	0.138439	0.4288
**_Single_hop_syge_ben_cm**	**KOOS_S**	74	0.02876	0.02877	0.0001970	0.02856	-0.201248	0.255396	0.8085
**__6_m_timed_hop_syge_ben**	**KOOS_S**	74	-0.08028	-0.08046	-0.0005499	-0.07974	-0.302721	0.151522	0.4978
**_Triple_hop_syge_ben**	**KOOS_S**	74	0.02363	0.02364	0.0001619	0.02347	-0.206134	0.250625	0.8421

Pearson Correlation Statistics (Fisher's z Transformation)

Variable	With Variable	N	Sample Correlation	Fisher's z	Bias Adjustment	Correlation Estimate	95% Confidence Limits		p Value for H0:Rho=0
**_Cross_over_hop_syge_ben**	**KOOS_S**	74	-0.10975	-0.11019	-0.0007517	-0.10900	-0.329302	0.122546	0.3532
**_Gennemsnit___i_forhold_til_rask**	**KOOS_S**	74	-0.08224	-0.08243	-0.0005633	-0.08168	-0.304498	0.149609	0.4873
**Rolimeter**	**KOOS_S**	74	-0.02534	-0.02535	-0.0001736	-0.02517	-0.252219	0.204504	0.8309
**Satisfaction**	**KOOS_S**	74	0.12839	0.12910	0.0008793	0.12752	-0.104009	0.345939	0.2767
**Repeat_surgery**	**KOOS_S**	74	0.10480	0.10518	0.0007178	0.10409	-0.127444	0.324859	0.3755
**_Single_hop_syge_ben_cm**	**KOOS_P**	74	0.20730	0.21034	0.00142	0.20594	-0.023676	0.414911	0.0763
**__6_m_timed_hop_syge_ben**	**KOOS_P**	74	0.13002	0.13076	0.0008905	0.12914	-0.102380	0.347388	0.2706
**_Triple_hop_syge_ben**	**KOOS_P**	74	0.29038	0.29899	0.00199	0.28856	0.064303	0.485076	0.0118
**_Cross_over_hop_syge_ben**	**KOOS_P**	74	0.09435	0.09463	0.0006462	0.09371	-0.137738	0.315454	0.4252
**_Gennemsnit___i_forhold_til_rask**	**KOOS_P**	74	0.37527	0.39454	0.00257	0.37306	0.158030	0.554305	0.0009

Pearson Correlation Statistics (Fisher's z Transformation)

Variable	With Variable	N	Sample Correlation	Fisher's z	Bias Adjustment	Correlation Estimate	95% Confidence Limits		p Value for H0:Rho=0
**Rolimeter**	**KOOS_P**	74	0.12900	0.12972	0.0008836	0.12813	-0.103395	0.346485	0.2744
**Satisfaction**	**KOOS_P**	74	0.54898	0.61692	0.00376	0.54634	0.363185	0.688848	<.0001
**Repeat_surgery**	**KOOS_P**	74	0.16954	0.17119	0.00116	0.16841	-0.062495	0.382200	0.1492
**_Single_hop_syge_ben_cm**	**KOOS_A**	74	0.21577	0.21922	0.00148	0.21436	-0.014865	0.422182	0.0647
**__6_m_timed_hop_syge_ben**	**KOOS_A**	74	0.10210	0.10246	0.0006993	0.10141	-0.130101	0.322439	0.3879
**_Triple_hop_syge_ben**	**KOOS_A**	74	0.25091	0.25638	0.00172	0.24930	0.022054	0.452045	0.0307
**_Cross_over_hop_syge_ben**	**KOOS_A**	74	0.06009	0.06017	0.0004116	0.05968	-0.171148	0.284306	0.6122
**_Gennemsnit___i_forhold_til_rask**	**KOOS_A**	74	0.42354	0.45200	0.00290	0.42116	0.213171	0.592624	0.0001
**Rolimeter**	**KOOS_A**	74	0.06366	0.06375	0.0004360	0.06323	-0.167696	0.287570	0.5912
**Satisfaction**	**KOOS_A**	74	0.41751	0.44467	0.00286	0.41515	0.206210	0.587880	0.0002
**Repeat_surgery**	**KOOS_A**	74	0.10080	0.10115	0.0006904	0.10012	-0.131385	0.321269	0.3941

Pearson Correlation Statistics (Fisher's z Transformation)

Variable	With Variable	N	Sample Correlation	Fisher's z	Bias Adjustment	Correlation Estimate	95% Confidence Limits		p Value for H0:Rho=0
**_Single_hop_syge_ben_cm**	**KOOS_SP**	74	0.25908	0.26512	0.00177	0.25742	0.030729	0.458925	0.0255
**__6_m_timed_hop_syge_ben**	**KOOS_SP**	74	0.16024	0.16163	0.00110	0.15917	-0.071949	0.374061	0.1732
**_Triple_hop_syge_ben**	**KOOS_SP**	74	0.26416	0.27057	0.00181	0.26247	0.036140	0.463191	0.0226
**_Cross_over_hop_syge_ben**	**KOOS_SP**	74	0.18847	0.19075	0.00129	0.18722	-0.043122	0.398665	0.1080
**_Gennemsnit___i_forhold_til_rask**	**KOOS_SP**	74	0.41295	0.43916	0.00283	0.41060	0.200959	0.584283	0.0002
**Rolimeter**	**KOOS_SP**	74	0.11514	0.11566	0.0007887	0.11437	-0.117196	0.334132	0.3298
**Satisfaction**	**KOOS_SP**	74	0.51226	0.56579	0.00351	0.50967	0.318226	0.661166	<.0001
**Repeat_surgery**	**KOOS_SP**	74	0.11579	0.11631	0.0007931	0.11500	-0.116557	0.334708	0.3271
**_Single_hop_syge_ben_cm**	**KOOS_Q**	74	0.28050	0.28822	0.00192	0.27873	0.053645	0.476855	0.0152
**__6_m_timed_hop_syge_ben**	**KOOS_Q**	74	0.17559	0.17743	0.00120	0.17443	-0.056315	0.387484	0.1349
**_Triple_hop_syge_ben**	**KOOS_Q**	74	0.28991	0.29847	0.00199	0.28809	0.063789	0.484682	0.0119

Pearson Correlation Statistics (Fisher's z Transformation)

Variable	With Variable	N	Sample Correlation	Fisher's z	Bias Adjustment	Correlation Estimate	95% Confidence Limits		p Value for H0:Rho=0
**_Cross_over_hop_syge_ben**	**KOOS_Q**	74	0.14913	0.15025	0.00102	0.14813	-0.083181	0.364301	0.2055
**_Gennemsnit___i_forhold_til_rask**	**KOOS_Q**	74	0.39917	0.42266	0.00273	0.39687	0.185162	0.573372	0.0004
**Rolimeter**	**KOOS_Q**	74	0.08688	0.08710	0.0005951	0.08629	-0.145067	0.308704	0.4630
**Satisfaction**	**KOOS_Q**	74	0.52432	0.58228	0.00359	0.52171	0.332901	0.670305	<.0001
**Repeat_surgery**	**KOOS_Q**	74	0.10747	0.10788	0.0007361	0.10674	-0.124803	0.327256	0.3633

confidence intervals computed based on Fishers z-transformation

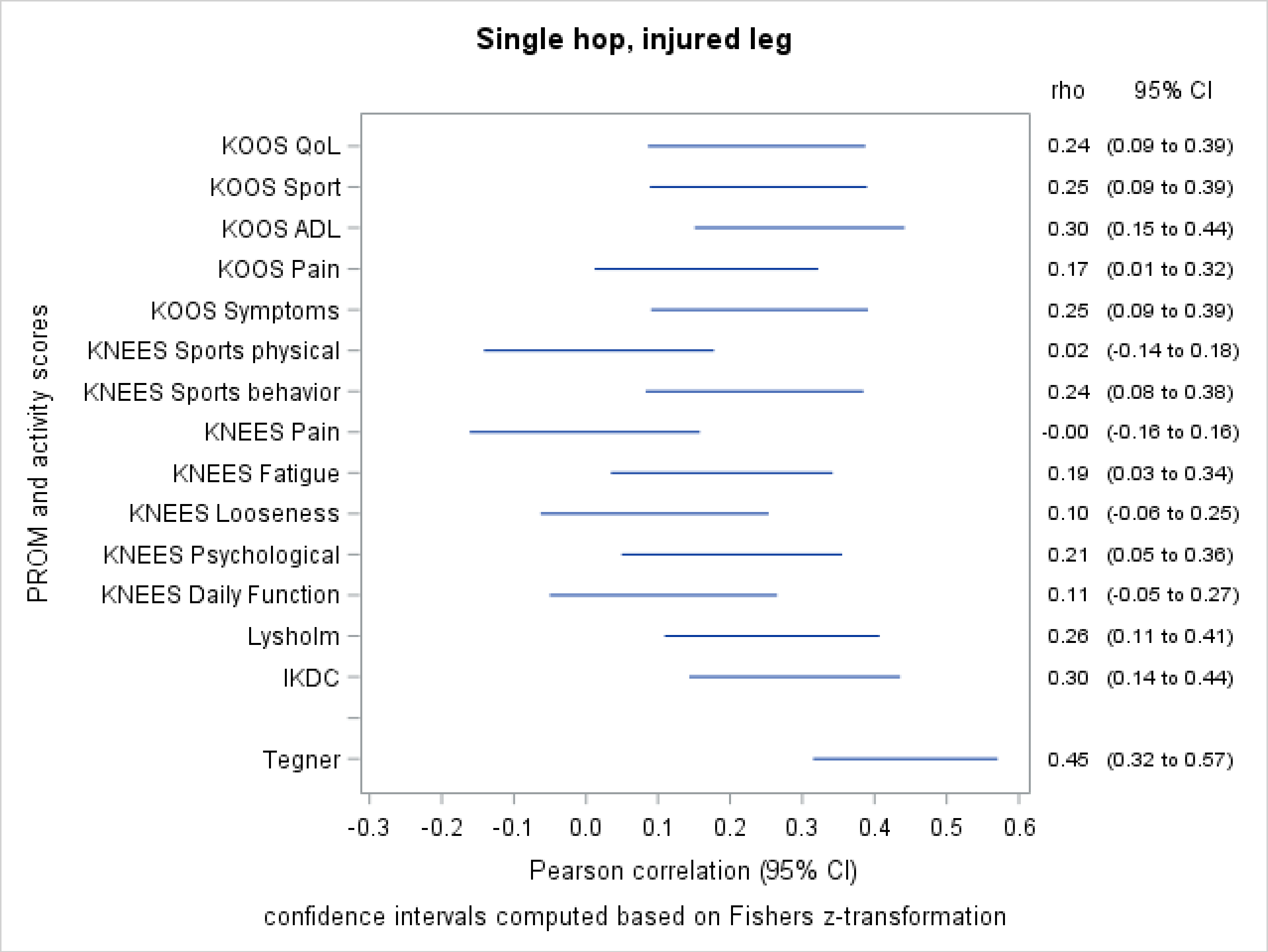
Single hop, injured leg
rho
95% CI
KOOS QoL
KOOS Sport
KOOS ADL
KOOS Pain
KOOS Symptoms
KNEES Sports physical
KNEES Sports behavior
KNEES Pain
KNEES Fatigue
KNEES Looseness
KNEES Psychological
KNEES Daily Function
Lysholm
IKDC
Tegner
PROM and activity scores
0.24 (0.09 to 0.39)
0.25 (0.09 to 0.39)
0.30 (0.15 to 0.44)
0.17 (0.01 to 0.32)
0.25 (0.09 to 0.39)
0.02 (-0.14 to 0.18)
0.24 (0.08 to 0.38)
-0.00 (-0.16 to 0.16)
0.19 (0.03 to 0.34)
0.10 (-0.06 to 0.25)
0.21 (0.05 to 0.36)
0.11 (-0.05 to 0.27)
0.26 (0.11 to 0.41)
0.30 (0.14 to 0.44)
0.45 (0.32 to 0.57)
-0.3 -0.2 -0.1 0.0 0.1 0.2 0.3 0.4 0.5 0.6
Pearson correlation (95% CI)
confidence intervals computed based on Fishers z-transformation

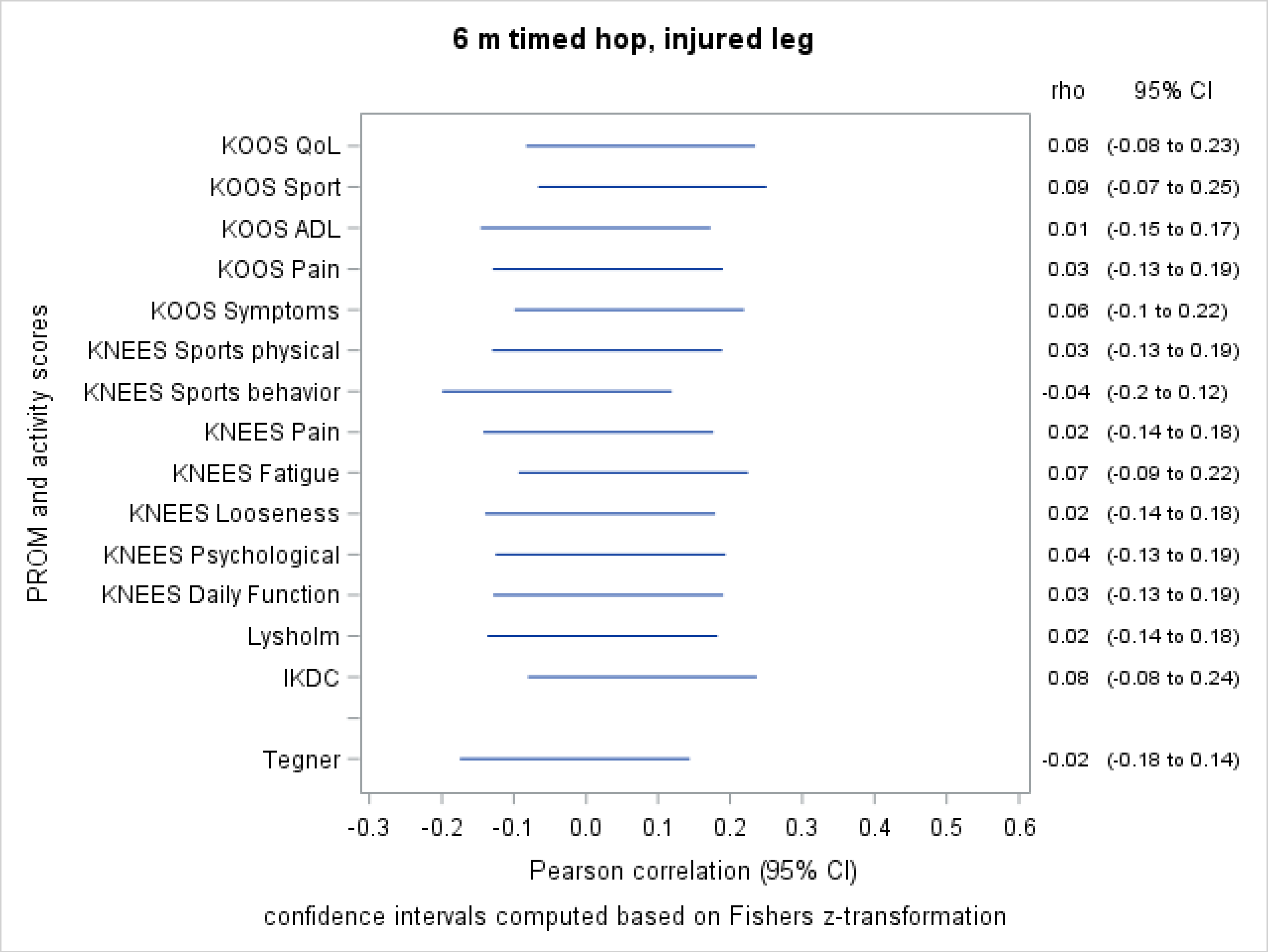

6 m timed hop, injured leg
rho
95% CI
KOOS QoL
0.08 (-0.08 to 0.23)
KOOS Sport
0.09 (-0.07 to 0.25)
KOOS ADL
0.01 (-0.15 to 0.17)
KOOS Pain
0.03 (-0.13 to 0.19)
KOOS Symptoms
0.06 (-0.1 to 0.22)
KNEES Sports physical
0.03 (-0.13 to 0.19)
KNEES Sports behavior
-0.04 (-0.2 to 0.12)
KNEES Pain
0.02 (-0.14 to 0.18)
KNEES Fatigue
0.07 (-0.09 to 0.22)
KNEES Looseness
0.02 (-0.14 to 0.18)
KNEES Psychological
0.04 (-0.13 to 0.19)
KNEES Daily Function
0.03 (-0.13 to 0.19)
Lysholm
0.02 (-0.14 to 0.18)
IKDC
0.08 (-0.08 to 0.24)
Tegner
-0.02 (-0.18 to 0.14)
PROM and activity scores
-0.3 -0.2 -0.1 0.0 0.1 0.2 0.3 0.4 0.5 0.6
Pearson correlation (95% CI)
confidence intervals computed based on Fishers z-transformation

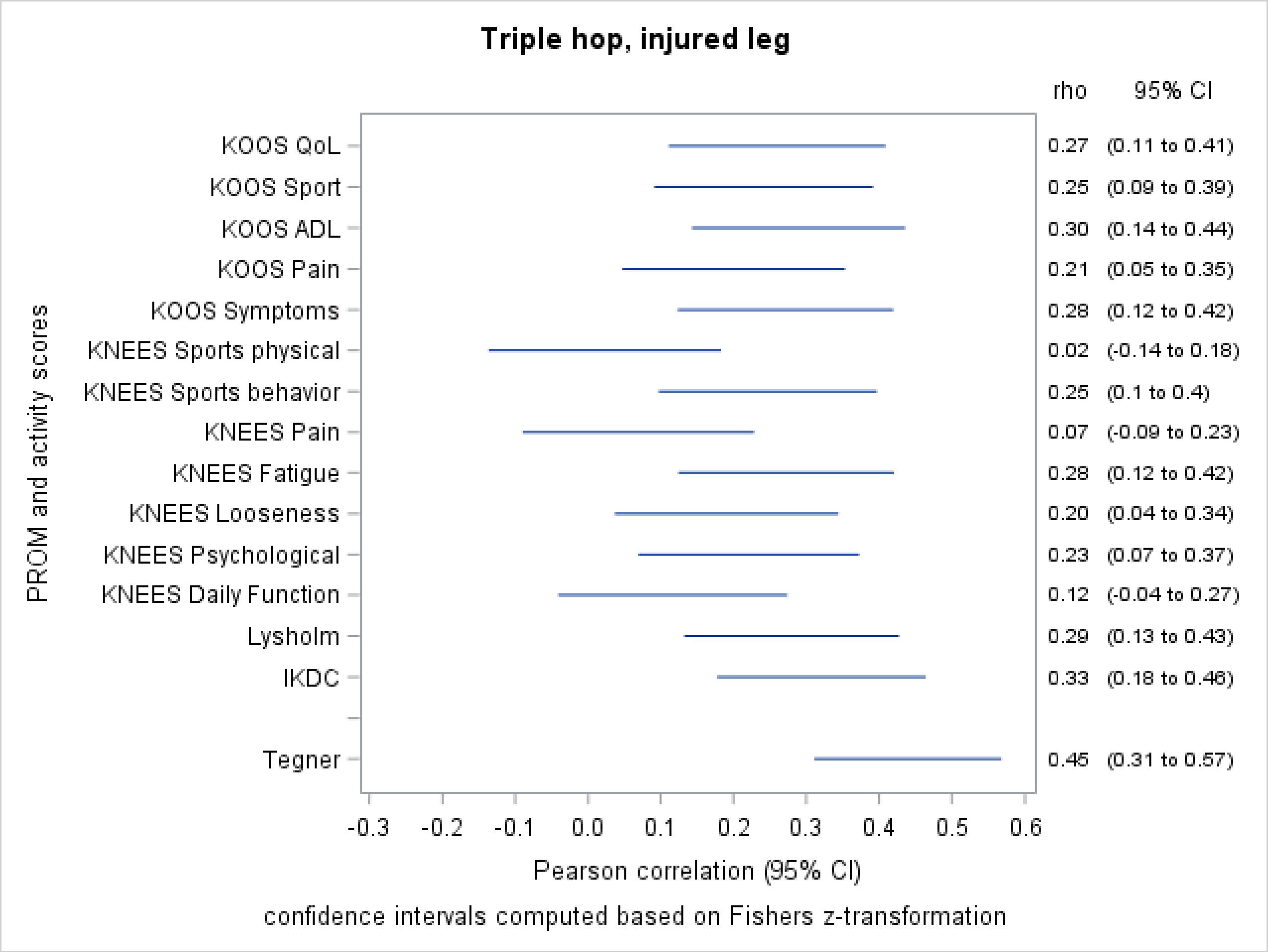
Triple hop, injured leg
rho
95% CI
KOOS QoL
KOOS Sport
KOOS ADL
KOOS Pain
KOOS Symptoms
KNEES Sports physical
KNEES Sports behavior
KNEES Pain
KNEES Fatigue
KNEES Looseness
KNEES Psychological
KNEES Daily Function
Lysholm
IKDC
Tegner
0.27 (0.11 to 0.41)
0.25 (0.09 to 0.39)
0.30 (0.14 to 0.44)
0.21 (0.05 to 0.35)
0.28 (0.12 to 0.42)
0.02 (-0.14 to 0.18)
0.25 (0.1 to 0.4)
0.07 (-0.09 to 0.23)
0.28 (0.12 to 0.42)
0.20 (0.04 to 0.34)
0.23 (0.07 to 0.37)
0.12 (-0.04 to 0.27)
0.29 (0.13 to 0.43)
0.33 (0.18 to 0.46)
0.45 (0.31 to 0.57)
PROM and activity scores
-0.3 -0.2 -0.1 0.0 0.1 0.2 0.3 0.4 0.5 0.6
Pearson correlation (95% CI)
confidence intervals computed based on Fishers z-transformation
